# Lake Superior Has Lost over 90% of Its Pesticide HCH Load since 1986

**DOI:** 10.1021/acs.est.0c07549

**Published:** 2021-04-07

**Authors:** Terry F. Bidleman, Sean Backus, Alice Dove, Rainer Lohmann, Derek Muir, Camilla Teixeira, Liisa Jantunen

**Affiliations:** † Department of Chemistry, Umeå University, Umeå, SE-90187, Sweden; ‡ Great Lakes Ecosystem Management Section, Environment and Climate Change Canada, Burlington, Ontario L7R 4A6, Canada; § Water Quality Monitoring and Surveillance Division, Environment and Climate Change Canada, Burlington, Ontario L7R 4A6, Canada; ∥ Graduate School of Oceanography, University of Rhode Island, Narragansett, Rhode Island 02882, United States; ⊥ Aquatic Contaminants Research Division, Environment and Climate Change Canada, Burlington, Ontario L7R 4A6, Canada; # Air Quality Processes Research Section, Environment and Climate Change Canada, Egbert, Ontario L0L 1N0, Canada

## Abstract

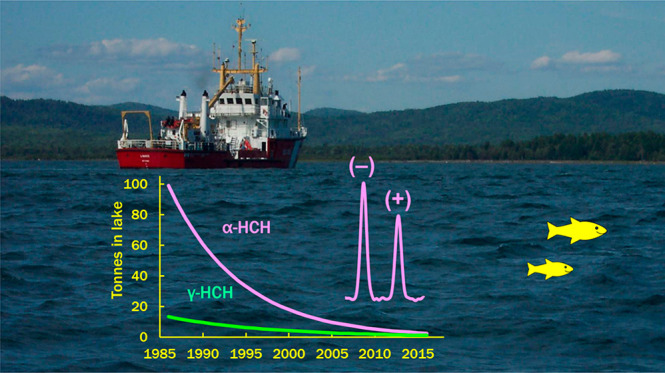

The time trend of α- and γ-hexachlorocyclohexane (HCH) isomers in Lake Superior water was followed from 1986 to 2016, the longest record for any persistent organic pollutant (POP) in Great Lakes water. Dissipation of α-HCH and γ-HCHs was first order, with halving times (*t*_1/2_) of 5.7 and 8.5 y, respectively. Loss rates were not significantly different starting a decade later (1996–2016). Concentrations of β-HCH were followed from 1996–2016 and dissipated more slowly (*t*_1/2_ = 16 y). In 1986, the lake contained an estimated 98.8 tonnes of α-HCH and 13.2 tonnes of γ-HCH; by 2016, only 2.7% and 7.9% of 1986 quantities remained. Halving times of both isomers in water were longer than those reported in air, and for γ-HCH, they were longer in water than those reported in lake trout. Microbial degradation was evident by enantioselective depletion of (+)α-HCH, which increased from 1996 to 2011. Volatilization was the main removal process for both isomers, followed by degradation (hydrolytic and microbial) and outflow through the St. Mary’s River. Sedimentation was minor. Major uncertainties in quantifying removal processes were in the two-film model for predicting volatilization and in microbial degradation rates. The study highlights the value of long-term monitoring of chemicals in water to interpreting removal processes and trends in biota.

## 1 Introduction

The five Laurentian Great Lakes bordering Canada and the United States have been recipients of persistent and toxic chemicals for many decades. Inputs came through direct discharge and runoff from the watersheds, but were often dominated by atmospheric deposition, especially for the larger lakes.^1−3^ The Great Lakes have responded rapidly to declines in atmospheric concentrations of persistent organic pollutants (POPs), such as polychlorinated biphenyls (PCBs) and organochlorine pesticides (OCPs),^4−6^ and burdens in fish have also decreased.^7−9^ Over time, the lakes have shifted from being net recipients to “secondary sources” of some POPs due to revolatilization from lake water.^4,6^ Since the early 1990s, The Canada-U.S. Integrated Atmospheric Deposition Network (IADN) and the Canadian Great Lakes Basin Monitoring and Surveillance Network (GLB) have monitored temporal trends of POPs and other chemicals of emerging concern in Great Lakes air and precipitation. Periodic reports have documented temporal trends in air concentrations, and atmospheric deposition/volatilization flows of toxic chemicals to and from the lakes. The most recent of these, which updates earlier reports, integrated a 20-year period from the early 1990s to 2012–2013 for air concentrations^4,5^ and up to 2012–2015 for atmospheric mass flows.^4,6^

Lake Superior (LS) is the largest of the Great Lakes: second in the world by area (82100 km^2^) and fourth by volume (12100 km^3^). LS is cold (mean temperature 5 °C), and has an average water retention time of 191 y.^10^ Atmospheric deposition and volatilization are major input and removal pathways for semivolatile chemicals.^1^ PCBs were lost from LS, mainly by volatilization, with a halving time (*t*_1/2_) of 3.5 y between 1978 and 1992.^11,12^ The OCP toxaphene increased in the water column of LS from 1950 to the mid-1970s and then incurred net loss by volatilization through the 1990s.^3,13^

Hexachlorocyclohexane (HCH) is one of many OCPs found in Great Lakes water and air. Technical HCH products contain several isomers, of which α-HCH is the most abundant, β-HCH is the most persistent and toxic, and γ-HCH is the only isomer with insecticidal activity.^14−16^ Technical HCH was discontinued in the U.S. and Canada in the 1970s and in some Asian countries during the 1980s and early 1990s,^15−17^ but formulations of pure γ-HCH (lindane) continued to be used worldwide through the 1990s and into the first decade of this century.^14,17−20^ Lindane was deregistered for agricultural and veterinary uses in phase-outs between 2001 and 2005 in Canada and 1998 and 2009 in the U.S.^21^ Global production and usage of technical HCH and lindane were stopped in 2009 under the Stockholm Convention, with exceptions for some pharmaceutical uses of lindane.^14^ Technical HCH was the starting product for the manufacture of lindane, which resulted in an estimated 6 to 7 million tonnes of “HCH waste” (consisting mainly of α-HCH, β-HCH, and δ-HCH) produced and discarded or stored in poorly managed sites around the world.^14^

HCH is the most abundant OCP in LS water, exceeding concentrations of toxaphene,^3,13,22,23^ chlordanes, DDTs, endosulfans, and dieldrin.^24,25^ Air concentrations of HCHs at Great Lakes monitoring stations have declined since the early 1990s, with halving times (*t*_1/2_) of about 4 to 5 y and only slight differences between the α-HCH and γ-HCH isomers and among the stations.^5,6^ Volatilization is a major loss process for HCHs,^4^ but it is unclear whether this is the only loss process. Variations in gas exchange direction and magnitude over time depend not only on the temporal trends in POPs atmospheric concentrations but also on their concentrations in lake water.^4,6^

In comparison to that of air, there has been less monitoring of POPs in Great Lakes water. Available data come from regular surveillance cruises and sporadic campaigns, but temporal trends in lake water are seldom reported. Lack of annual water concentration data limited the mass flow estimates for POPs in the Great Lakes.^4^ Here, we examine the time course of HCHs in LS water over 30 years (1986–2016) and evaluate processes which remove them from the lake. Our documentation is the longest period for any POP in Great Lakes water and provides an accurate record for interpreting time trends in biota.

## 2 Methods

The upper water column (“surface” water, ≤12 m) of LS was sampled for HCHs and other organic compounds in 11 years between 1986 and 2016. Collections were done mainly on spring (May, June) and summer (August) surveillance cruises which occupied the same stations each time, covered the nearshore and open lake areas (Supporting Information, Table SI-1.1, Figure SI-1.1), and occasionally included deep water (>12–270 m).^24^ The total number of surface measurements was 283 for α-HCH, 257 for γ-HCH, and 103 for β-HCH. Sampling methods varied. A submersible pump was used to take surface water onboard where it was stored in glass carboys or stainless steel cans.^24−26^ In some cases, water was collected in oceanographic bottles (e.g., Go-Flo) lined with polytetrafluoroethylene^24^ or sampled passively *in situ* with low-density polyethylene (LDPE) film.^27^ Whole water samples were examined in 1986–1987, while in other years only the dissolved phase was analyzed after excluding the particulate fraction by centrifugation, filtration or passive sampling, and isolating the dissolved fraction using large volume liquid extraction (LVX),^26^ resin cartridges,^24,25^ or solvent extraction of passive samplers.^27^ An earlier study found that HCHs were dissolved and not detectable on glass fiber filters which preceded resin cartridges.^28^ The same paper estimated the particulate fraction, considering sorption to particles and association with dissolved/colloidal organic matter, and concluded that 99% of the HCHs were dissolved. Analytical methods used capillary gas chromatography with electron capture detection (ECD), quadrupole mass spectrometry in the electron capture negative ion mode (ECNI-MS), electron impact MS/MS or high-resolution mass spectrometry. Sampling, extraction and analytical methods are summarized in Table SI-1.1, and quality control measures are documented.^24−27^ The two enantiomers of the chiral compound α-HCH were determined for water samples collected from 1996 to 1997 and in 2001, 2005, 2008, and 2011, using two GC columns with different chiral stationary phases which reversed the enantiomer elution order (SI-1).^24^

## 3 Results and Discussion

### 3.1 Concentrations and Proportions of HCH Isomers and α-HCH Enantiomers in LS Water

**Figure 1:**
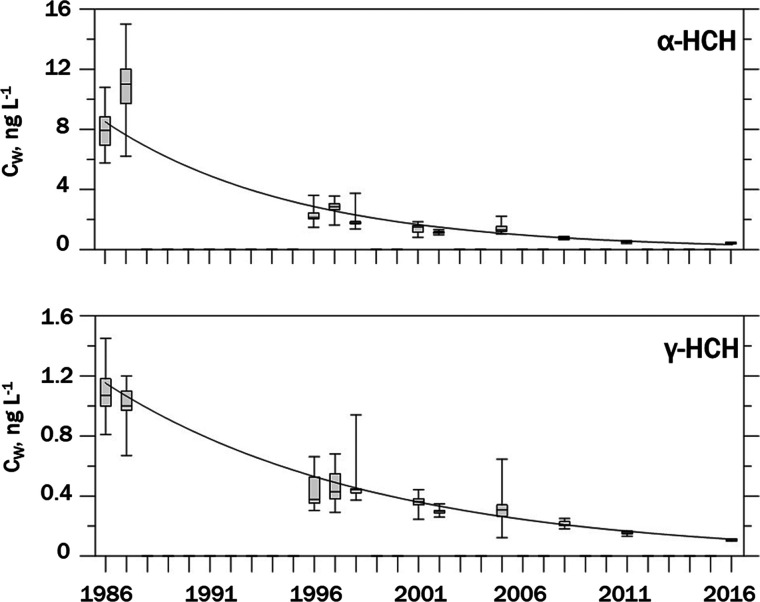
Decline in α-HCH (top) and γ-HCH (bottom) concentrations in LS water from 1986 to 2016. Whiskers show ranges of concentrations, boxes show quartiles with median as a horizontal line. Data are summarized in Table SI-1.1. Time trends are also displayed in Figure 3, Figure SI-1.2 and Figure SI-1.3.

**Figure 2:**
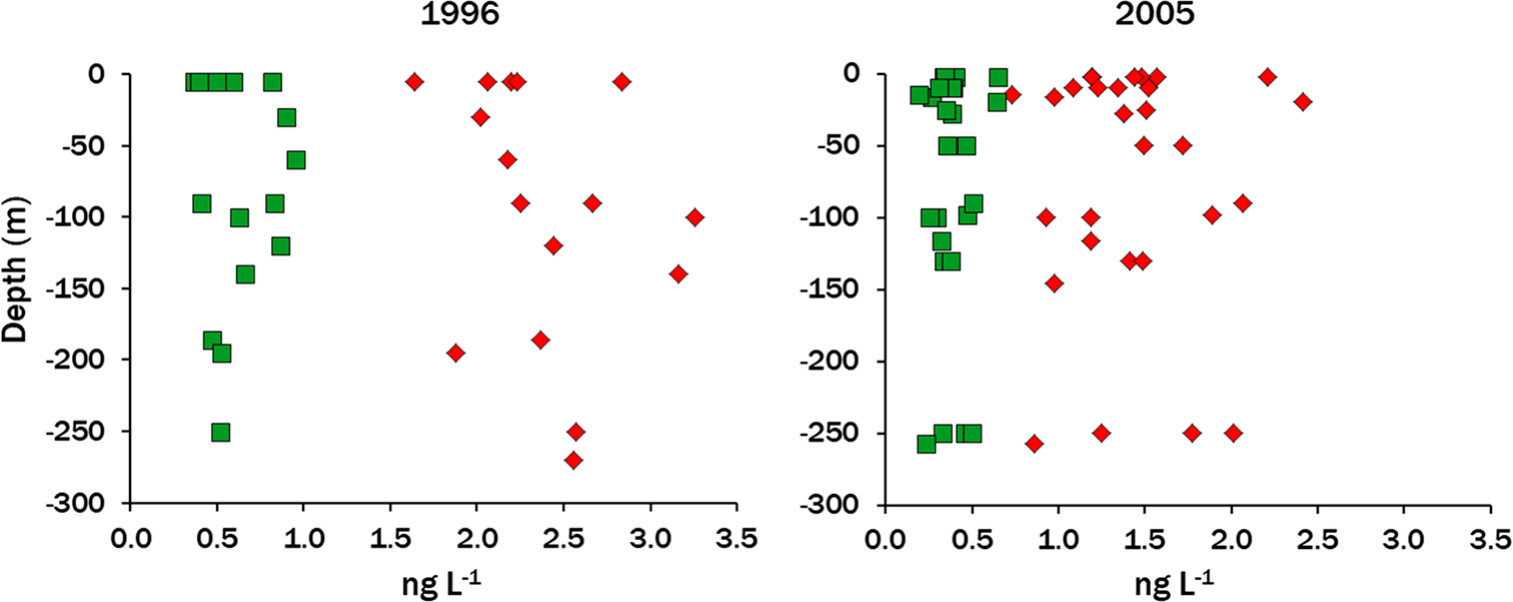
Depth profiles of α-HCH (red diamonds) and γ-HCH (green squares) in LS, August 1996 and August 2005. There are no significant depth trends for either isomer (*p* > 0.05).

Table 1 reports annual geometric mean (GM) concentrations of α-HCH, β-HCH, and γ-HCH in LS surface water. An expanded data table which includes results of multiple samplings within a year, arithmetic annual means, standard deviations, sample numbers, and collection/analysis methods is provided in Table SI-1.1. The decreasing trend in annual GM α-HCH and γ-HCH concentrations over the 30-year period is shown in Figure 1. Depth profiles were taken from the surface to 250–270 m in August 1996 and August 2005 and there was no significant trend with depth for either isomer (Figure 2). LS is dimictic and appears to be well mixed with respect to HCHs, at least to these depths.

**Table 1:** Annual Geometric Mean HCH Concentrations (ng L^–1^) and EFs of α-HCH in LS Surface Water^a^^,^^b^

Years	α-HCH	β-HCH	γ-HCH	EF of α-HCH	γ-HCH/α-HCH	β-HCH/α-HCH
2016	0.34	0.027	0.083		0.24	0.079
2011	0.21	0.036	0.13	0.413	0.62	0.171
2008	0.75		0.19	0.424	0.25	
2005	1.17	0.047	0.29	0.434	0.25	0.040
2002	1.13	0.042	0.30		0.26	0.037
2001	1.38		0.35		0.25	
1998	1.80	0.078	0.45	0.438	0.25	0.043
1997	2.52	0.063	0.44		0.17	0.025
1996	2.11	0.056	0.39	0.450	0.18	0.027
1987	10.8		1.01		0.09	
1986	7.88		1.08		0.14	

a Extended data in Table Sl-1.1: collection/analytical methods, multiple samples within a year, arithmetic means, standard deviations, number of samples, and data sources.

b Isomer ratios calculated from annual geometric means.

Isomer proportions in lake water changed over the study period (Table 1). The ratio of annual GM γ-HCH/α-HCH concentrations rose from 0.09 to 0.14 in the 1980s to 0.25 in 1998 and remained between 0.24 and 0.26 through 2016, with one aberrant value of 0.62 in 2011. The β-HCH/α-HCH ratio also increased since it was first measured in 1996, probably reflecting the greater environmental stability of β-HCH,^16^ and lower Henry’s law constant^29^ which disfavors volatilization. The increasing γ-HCH/α-HCH ratio fits with the historical shift in HCH product usage from technical HCH, containing 55–80% α-HCH, 8–15% γ-HCH, 5–14% β-HCH, and other isomers to formulations of lindane (γ-HCH).^16,18^ Technical HCH was discontinued in Canada in 1971 and the U.S. in 1978,^16^ and lindane was used in both countries until registrations for agricultural and veterinary applications were canceled in 2004 and 2009.^21^

Passive air samplers were deployed across North America in 2000–2001 to determine the continent-wide distribution of HCHs in the atmosphere.^30^ Ratios of γ-HCH/α-HCH varied from 0.07–5, with a continental average of 1.0. Regions with lower ratios, Atlantic and Pacific coasts of Canada, eastern Canadian Arctic, and Canadian mountains, were likely influenced by long-range transport from outside North America and in coastal areas to revolatilization of α-HCH from seawater. Higher ratios were found in the Canadian prairies, the eastern U.S.A., and Mexico, which were more influenced by lindane usage. Compared to those of the latter group, ratios near the Great Lakes were slightly lower and were attributed to preferential of volatilization α-HCH from Great Lakes water.^30^

The enantiomer fraction (EF) was calculated from concentrations of the (+) and (−) enantiomers.^31^
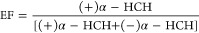
1

Mean EFs in surface water ranged from 0.450 ± 0.005 in 1996 to 0.413 ± 0.002 in 2011 (Table 1) and decreased linearly over 15 years with r^2^ = 0.94 (Figure SI-1.4). Decreasing EFs indicate greater microbial degradation of (+)α-HCH over time. Depth profiles taken in August 2005 (Figure SI-1.4) show no variation in EF with depth which is strikingly different from the strongly decreasing gradient in EFs observed for the stratified Arctic Ocean,^32−36^ and those together with invariant α-HCH concentrations with depth (Figure 2) indicate a well-mixed lake. Revolatilization of microbially degraded α-HCH that was depleted in the (+) enantiomer lowered EFs in air over and near the Great Lakes,^24,30,37^ the Atlantic coast of Canada,^30^ and the eastern Canadian Arctic.^30,34,35,38^

### 3.2 Time Trends of HCHs in Water, Air and Fish

**Figure 3:**
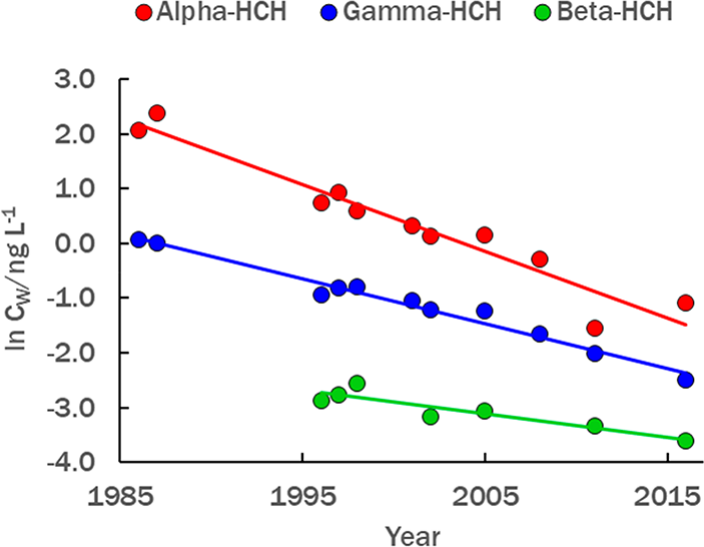
First-order decline in annual geometric mean concentrations of α-HCH, β-HCH, and γ-HCH; regression equations: ln *C*_W_ (α-HCH) = 244.3918–0.122*year; ln *C*_W_ (β-HCH) = 82.279–0.0426*year; ln *C*_W_ (γ-HCH) = 162.8215–0.08194*year. Regressions for arithmetic means and all data points are shown in Figures SI-1.2 and SI-1.3.

Dissipation of HCHs from LS by all processes (F_LOSS_, kg y^–1^) was followed by plotting the natural logarithm of annual GM water concentrations (C_W,_ ng L^–1^) vs year (Table 1, Figure 3). Similar plots using annual arithmetic mean (AM) concentrations and all concentration points for all years are shown in Figures SI-1.2 and SI-1.3. Table 2 presents first-order dissipation rate constants (k_DISS_, y^–1^), halving times (*t*_1/2_, y = 0.693/k_DISS_), and 95% confidence intervals (CI = ± t_0.05, n-2)_*SE), where SE is the standard error of k_DISS_.^5^ The *t*_1/2_ values (±95% CI) derived from GM regression in the time series 1986–2016 (Water 1) were α-HCH 5.68 y (4.71–7.16 y) and γ-HCH 8.46 y (7.55–9.62 y). Faster loss was found for 25 PCB congeners between 1980 and 1992, with *t*_1/2_ = 3.5 y.^12^

**Table 2:** Dissipation Rate Constants (k_DISS_) and Halving Times (*t*_1/2_) of HCHs in Lake Superior Water^a^

			Water 1, 1986 to 2016							Water 2, 1996 to 2016				
Regression method^b^	r^2^	k_DISS, _y^–1^	–95% Cl	+95% Cl	t_1/2_, y	–95% Cl	+95% Cl	r^2^	k_DISS, _y^–1^	–95% Cl	+95% Cl	t_1/2_, y	–95% Cl	+95% Cl
α-HCH														
AM	0.95	0.116	0.136	0.0970	5.96	5.11	7.15	0.91	0.105	0.134	0.0761	6.61	5.19	9.11
GM	0.93	0.122	0.147	0.0968	5.68	4.71	7.16	0.86	0.113	0.155	0.0717	6.11	4.47	9.67
All	0.73	0.130	0.140	0.121	5.32	4.97	5.73	0.57	0.130	0.145	0.116	5.31	4.79	5.97
β-HCH														
AM								0.77	0.0393	0.0640	0.0146	17.6	10.8	47.5
GM								0.83	0.0426	0.0650	0.0201	16.3	10.7	34.4
All								0.28	0.0205	0.0269	0.0141	33.8	25.7	49.3
γ-HCH														
AM	0.97	0.0795	0.0896	0.0694	8.72	7.73	9.98	0.94	0.0798	0.0980	0.0616	8.69	7.07	11.3
GM	0.98	0.0819	0.0918	0.0721	8.46	7.55	9.62	9.95	0.0833	0.101	0.0655	8.32	6.86	10.6
All	0.75	0.0771	0.0826	0.0716	8.99	8.39	9.68	0.57	0.0748	0.0835	0.0661	9.27	8.30	10.5

a k_DISS_ = slope of ln C_w_/ng L^–1^ vs year, *t*_1/2_ = 0.693/k_DISS_

b Regression of natural logarithms of C_w_/ng L^–1^: AM = annual arithmetic means, GM = annual geometric means, All = all data points.

The gap between 1986 and 1987 and later measurements prompted us to examine the shorter series from 1996 to 2016 (Water 2). Regressions of β-HCH from 1996 to 2016 were included in this set. The *t*_1/2_ values (±95% CI), derived from GM regressions for Water 2, were α-HCH 6.11 y (4.47–9.67 y), β-HCH 16.3 y (10.7–34.4 y), and γ-HCH 8.32 y (6.86–10.6 y). The 95% CIs for Water 1 and Water 2 overlapped, indicating similar *t*_1/2_ in the longer and shorter time series. The slower dissipation of β-HCH reflects its greater environmental persistence.^15,16^

Halving times of gas-phase HCHs in air at Eagle Harbor, LS, derived from annual GM concentrations monitored from 1991 to 2013 (Air 1, n = 19), were 4.29 ± 0.16 y for α-HCH and 4.55 ± 0.25 y for γ-HCH,^5^ and the 95% CI were 3.95–4.63 y and 4.03–5.07 y. Similar statistics for a shorter time interval (Air 2, 1999–2010, n = 12) were α-HCH: 3.92 ± 0.32 y (3.22–4.62 y) and γ-HCH: 3.09 ± 0.22 y (2.61–3.57 y).^9^ The 95% CI for both isomers in Air 1 were shorter than, and did not overlap, the 95% CI for Water 1 (Table 2), indicating slower dissipation in lake water. There was overlap of the 95% CI for α-HCH, but not γ-HCH, in Water 2 and Air 2.

HCHs in Superior lake trout (*Salvelinus namaycush*) declined from 1999 to 2010, with *t*_1/2_ 4.90 ± 0.83 y (95% CI 3.05–6.75 y) for α-HCH (n = 12) and 3.23 ± 0.92 y (95% CI 0.98–5.48 y) for γ-HCH (n = 8).^9^ The central *t*_1/2_ of both isomers are shorter for fish than those for Water 2 (1996 to 2016). The 95% CIs for fish and Water 2 overlap for α-HCH but not γ-HCH.

Monitoring data for lake trout, collected from the five Great Lakes during 1970s and 1980s and 2003, showed long-term declines in PCBs, polybrominated diphenyl ethers (PBDEs) and several OCPs (DDT compounds, dieldrin, chlordane compounds, and toxaphene), but rates of decline varied according to the compound, lake, and period of time over which k_DISS_ was calculated.^7^ In general, rates derived from 1970s and 1980s data were faster than those derived from monitoring in the 1980s and 2003, and some compounds showed net accumulation in the early time period. The authors stated that “As concentrations in fish reflect concentrations in water, the change in source functions could be the primary factor behind rate changes observed in fish.” Such changes were judged a more likely explanation for the observed rate changes, rather than changes in climate, food webs, or fisheries dynamics. Long-term data series for LS which spanned about 40 years for fish and 25 years for air showed close coupling of the decline rates in fish and air for PCBs and DDTs.^8^

The loss budgets of α-HCH and γ-HCH, calculated from GM concentrations, are reported in SI-2.1, Tables SI-2.1a,b. In 1986, LS contained 98700 kg of α-HCH and 13200 kg of γ-HCH, and between 1986 and 2016, it contained 90400 kg of α-HCH and 12200 kg of γ-HCH were dissipated (Table SI-2.1a,b). By the end of 2016, quantities of these two isomers remaining in the lake were only 2.7% and 7.9% of those in 1986.

Under the Canadian Environmental Protection Act,^39^ “Virtual Elimination is the ultimate reduction of the quantity or concentration of a toxic substance in the release into the environment below concentrations that can be accurately measured or the “level of quantification”. A similar concept defines “temporal environmental hysteresis” as the “time lag between when a pollutant’s input to the environment stops and when its concentration in the environment drops to some desired fraction of its maximum concentration.”^40^

The level of quantification (LOQ) is the lowest concentration of the toxic substance that can be accurately measured using sensitive but routine sampling and analytical methods.”^39^ Field blanks based on resin cartridge sampling of 80–100 L were 0.001 ng L^–1^ or lower (Table SI-1.1). With taking the LOQ as 10 times this level (0.01 ng L^–1^) and using the regression equations in Figure 3, extrapolated years of virtual elimination for the three HCHs in LS are 2040–2043.

### 3.3 HCH Removal Processes

F_LOSS_ is the net loss. HCHs have been continuously entering the lake at a declining rate, mainly by atmospheric deposition (gas exchange and precipitation). Atmospheric deposition of α-HCH exceeded volatilization until the mid-1990s; volatilization dominated through the 2000s, and between 2010 and 2015, the two flows were nearly even.^4^ The picture was similar for γ-HCH with different timing. Deposition exceeded volatilization through the early 2000s, after which volatilization became dominant and remained so through 2015.

F_LOSS_ is the sum of several loss processes: volatilization (F_VOL_), outflow through the St. Mary’s River (F_OUT_), sedimentation (F_SED_), and degradation due to basic hydrolysis (F_HYD_) and microbial breakdown (F_MIC_).
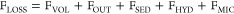
2

These dissipation processes are summarized below and in SI-2. The quantities of α-HCH and γ-HCH in the lake at the beginning of each year were subjected to the individual loss processes (eq 2), and their sum was compared to the directly determined F_LOSS_. The rate constants for F_VOL_, F_HYD_, and F_MIC_ were applied to the annually declining GM C_W_ (Figure 3). F_OUT_ and F_SED_ were calculated differently, as described below.

#### 3.3.1 Volatilization

We calculated F_VOL_ from the Whitman two-film gas exchange model in the form^4^
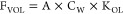
3where A is the area of LS (8.21 × 10^10^ m^2^), C_W_ (kg m^–3^) is the concentration in surface water, and K_OL_ (m y^–1^) is the annually averaged overall mass transfer coefficient, considered from the water side, which includes resistances to transfer in the water and air phases.^4,41^ Details are provided in SI-2.2 and Table SI-2.3. We used the smoothed record of annual GM concentrations in surface water from 1986 to 2016 (Figure 3, Table 1, Table SI-2.2) and assumed thermodynamically consistent “final adjusted values” (FAVs) for the Henry’s law constants of the HCHs.^29^ K_OL_ values were calculated at the high and low excursions of annual water temperature (273–293 K) and wind speed (4–8 m s^–1^), taking these excursions from Figure S2 in the Supporting Information of Guo et al.^4^ K_OL_ ranged from 4.8 to 44 m y^–1^ (GM 14 m y^–1^) for α-HCH and 2.0 to 19 m y^–1^ (GM 6.1 m y^–1^) for γ-HCH (Table SI-2.3). GM K_OL_ were used to calculate annual F_VOL_, which for α-HCH ranged from 9580 kg y^–1^ in 1986 to 247 kg y^–1^ in 2016 and totaled 81600 kg over 30 years (Table SI-2.1a). The total quantity of γ-HCH removed by volatilization ranged from 548 kg in 1986 to 47 kg in 2016 and totaled 6420 kg over 30 years.(Table SI-2.1b). These quantities are 90% and 53% of total measured F_LOSS_.

#### 3.3.2 Outflow

Outflow of HCHs from LS to Lake Huron takes place through the St. Mary’s River. Rather than using a single process parameter for outflow, annual smoothed HCH concentrations (Figure 3) were multiplied by annually averaged river discharge (Table SI-2.4) to obtain F_OUT_. Outflow removed 4570 kg of α-HCH and 830 kg of γ-HCH between 1986 and 2016, 5.1% and 6.8% of total measured F_LOSS_ (SI-2.3, Table SI-2.1a,b).

#### 3.3.3 Sedimentation

Few measurements were found for HCHs in LS sediments. We combined sediment concentration data from Jackfish Bay, averaged from 1986 and 1998 (SI-2.5, Table SI-2.5), with a high-end sedimentation accumulation rate (1 g m^–2^ d^–1^) (Table SI-2.5) to estimate F_SED_ of 2.6 and 2.1 kg y^–1^ for α-HCH and γ-HCH. Sediment accumulation of HCHs is a small term. Assuming the same F_SED_ each year, total removal over 30 years was 78 kg of α-HCH and 63 kg of γ-HCH. Even though sedimentation of HCHs is probably low, the sediments could participate in the geochemical cycling. For example, particle settling brings PCBs and other hydrophobic organic contaminants to the bottom of LS, but very little of this material is accumulated in the sediments. Instead, these compounds are efficiently “recycled” within the benthic nepheloid layer (BNL) by decomposition of the settling particulate organic matter and/or surficial sediments.^42−45^ If BNL recycling also occurs for HCHs, microbial processes in this layer might contribute to the enantioselective degradation of α-HCH observed in surface and deep water (Section 3.3.5).

#### 3.3.4 Hydrolysis

The α-HCH and γ-HCH isomers are subject to basic hydrolysis in the slightly alkaline (pH 7.83) water of LS (SI-2.5), whereas β-HCH is stable. We used the temperature-dependent second-order basic hydrolysis rate constants (k_B_, M^–1^ y^–1^) of Ngabe et al.^46^ to derive the pseudo first-order rate constants (k’_α_ = 0.0177 y^–1^; k’_γ_ = 0.0109 y^–1^) at pH 7.83 and 5 °C (SI-2.6, Table SI-2.6). Hydrolysis is a substantial portion (14–16%) of total measured F_LOSS_, accounting for 30-year removal of 14750 kg of α-HCH and 1670 kg of γ-HCH (Tables SI-2.1a,b)

#### 3.3.5 Microbial degradation

Aerobic microbial degradation of HCHs is common in soils, freshwater, groundwater, and seawater.^24,32−38,47−49^ Pathways for bacterial degradation of anthropogenic contaminants in soils have been extensively studied and involve several genera of Sphingomonad bacteria belonging to the class *Alphaproteobacteria* and family *Sphingomonadaceae*(49−53) as well as other degraders such as *Pseudomonas* spp.^51,54,55^ “Lin” enzymes in these bacteria catalyze degradation of HCHs. The initial conversion of α-HCH and γ-HCH to pentachlorocyclohexenes involves dehydrochlorinase LinA.^49,51,56,57^ Enzyme LinB catalyzes degradation of α-HCH, β-HCH, and δ-HCH to pentachlorocyclohexanols as a first step.^50,53,56^ LinA has two variants, LinA1 and LinA2, which dehydrochlorinate either the (+)α-HCH or (−)α-HCH enantiomer, respectively.^50−52,56,58^ Enantioselective fractionation of the two α-HCH enantiomers can be due to changes in the relative abundance and reactivity of LinA1 and LinA2 during bacterial growth.^51,52^

EFs of α-HCH in soil and water vary greatly according to selective degradation of either the (+) enantiomer (EF < 0.5) or the (−) enantiomer (EF > 0.5) (eq 1).^59,60^ It has been noted that enantioselective degradation of (+)α-HCH in fresh water tends to be favored in cold, oligotrophic systems, such as the Great Lakes and Arctic lakes, and less so in temperate lakes and wetlands.^47^ These findings led to the hypothesis that enantioselective degradation is optimized in nutrient-poor waters in which oligotrophic bacteria may act as biofilms.^47^ PCBs and other hydrophobic organic contaminants on settling particles are recycled within the BNL of LS by decomposition of the labile organic matter (Section 3.3.3).^42,44,45^ This may also provide an active environment for enantioselective degradation of α-HCH and other chiral compounds.

EFs of α-HCH in Lake Superior surface water declined linearly from 0.450 in 1996 to 0.413 in 2011 (r^2^ = 0.94), and in 2005, the EFs did not vary with depth (Table 1, Figure SI-1.4). From these results, the ratio of pseudo first-order microbial degradation rate constants was *k*_m+_/*k*_m–_ = 1.33 (Table SI-2.7). It is not possible to derive absolute rate constants from this ratio and the decline of total α-HCH (sum of enantiomers) because F_LOSS_ involves processes in addition to degradation.

Pseudo first-order microbial degradation rate constants (*k*_m_, y^–1^) for HCHs were reported in the Bering Sea–Eastern Arctic Ocean; *k*_m_ = 0.037 y^–1^ for γ-HCH, *k*_m+_ = 0.117 and *k*_m–_ = 0.030 y^–1^ for α-HCH enantiomers, and *k*_m_ = 0.117 + 0.030 = 0.147 y^–1^ for total α-HCH (sum of enantiomers).^32,33^ F_MIC_ for γ-HCH in LS was estimated using the Harner et al.^32,33^ rate constant, *k*_m_ = 0.037 y^–1^, which resulted in loss of 5620 kg over 30 years (Table SI-2.1b).

Two sets of microbial degradation estimates were made for α-HCH in LS. F_MIC1_ was predicted using *k*_m_ = 0.147 y^–1^ for total α-HCH.^32,33^ F_MIC2_ used *k*_m–_ = 0.030 y^–1^ and estimated *k*_m+_ = 0.040 y^–1^, based on our observed ratio of *k*_m+_/*k*_m–_ = 1.33 in LS. Thus, *k*_m_ for total α-HCH = *k*_m+_ + *k*_m–_ = 0.070 y^–1^ (SI-2, Figure SI-1.2, Table SI-1.4). Microbial degradation of α-HCH over 30 years totaled 115000 kg (F_MIC1_) or 56800 kg (F_MIC2_) (Table SI-2.1a).

Faster degradation has been estimated for total α-HCH in an Arctic lake^61,62^ and for α-HCH and γ-HCH in the Greenland Sea^63^ (Table SI-2.7), but the derived rate constants (*k*_m_ = 0.48–1.13 y^–1^) are too high for LS because they greatly exceed the rate constant for F_LOSS_ (0.122 y^–1^; Figure 3).

#### 3.3.6 Summary of Removal Processes

**Figure 4:**
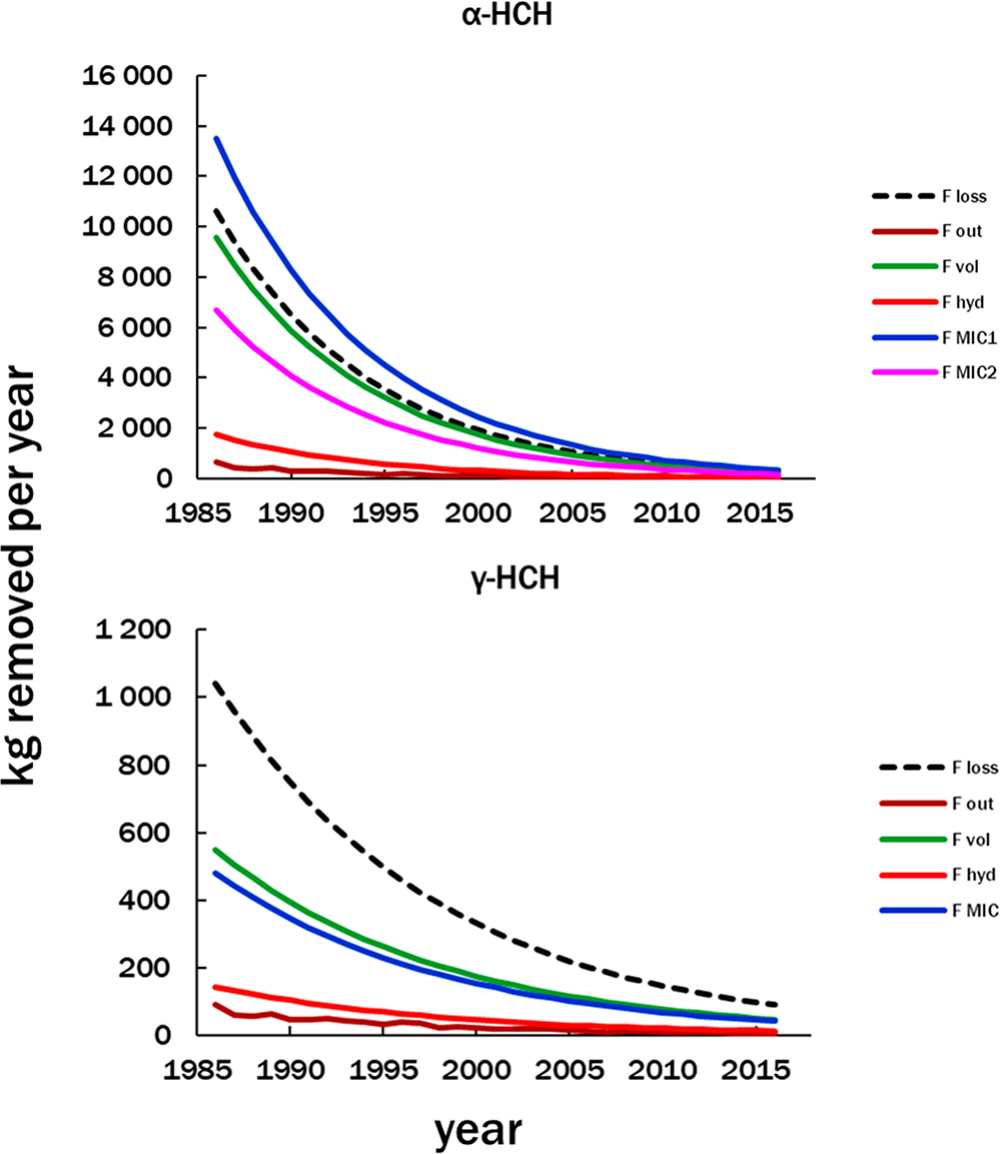
Annual removal flows (F_LOSS_, kg y^–1^) of HCHs over 30 years. F_LOSS_ is the decline of HCHs in the lake based on monitoring data (Figure 3). Other flows are estimated by applying process parameters to the declining annual concentrations: volatilization (F_VOL_), outflow (F_OUT_), hydrolysis (F_HYD_), and microbial degradation (F_MIC_). See corresponding sections here and in SI-2. The wavy lines for F_OUT_ are because calculations used annual discharges from the St. Mary’s river, which varied from year to year (Table SI-2.4). F_MIC_ for γ-HCH was estimated using the first-order microbial degradation rate constant reported in the Bering Sea – Eastern Arctic Ocean (*k*_m_ = 0.037 y^–1^).^32,33^ Two degradation rate constants were applied for total α-HCH (both enantiomers);^32,33^ F_MIC1_ used *k*_m_ = 0.147 y^–1^ and F_MIC2_ used *k*_m_ = 0.070 y^–1^, which was derived by considering the relative degradation rates of the two enantiomers in LS (SI-2, Figure SI-1.4, Table SI-2.7).

F_LOSS_ and component loss processes (eq 2) are summarized in Table SI-1.2a,b Totals of the annual F_LOSS_ give the quantities that dissipated over 30 years, 90400 kg of α-HCH and 12200 kg of γ-HCH. These measured losses can be compared with estimated losses due to individual processes, Table SI-1.2a,b and Figure 4. As noted in Section 3.3, rate constants for F_VOL_, F_HYD_, and F_MIC_ were applied to the annual GM C_W_; thus, their long-term rates are fixed by F_LOSS_ and magnitude by the rate constants of the individual processes. The “wavy” line for F_OUT_ in Figure 4 is because calculations used annual discharges from the St. Mary’s River, which varied from year to year. For γ-HCH, Process Sum 1 (F_VOL_ + F_OUT_ + F_HYD_) = 8920 kg or 73.2% of total F_LOSS_. The inclusion of microbial degradation (F_MIC_) brings Process Sum 2 (F_VOL_ + F_OUT_ + F_HYD_ + F_MIC_) to 14500 kg, which is 119% of total F_LOSS_. Thus, the process sums agree with the measured dissipation within about 20%.

The agreement is less satisfactory for α-HCH. Process Sum 1 (F_VOL_ + F_OUT_ + F_HYD_) = 101000 kg, or 112% of total measured F_LOSS_, 90400 kg, which would be good agreement except that it does not account for microbial degradation. Adding F_MIC1_ raises Process Sum 2 (F_VOL_ + F_OUT_ + F_HYD_ + F_MIC1_) to 239% of total F_LOSS_. Replacing F_MIC1_ with F_MIC2_, which uses a lower rate constant, results in Process Sum 3 (F_VOL_ + F_OUT_ + F_HYD_ + F_MIC2_) = 174% of total F_LOSS_. The comparisons of processes with F_LOSS_ are displayed in Figure 4.

Uncertainties in the loss budgets are discussed in SI-2.7. The 95% confidence intervals^5^ (CI) for k_DISS_ were derived from the standard error (SE) by t_0.05, n-2_*SE and are reported in Table 2 of the main paper. Corresponding halving times, calculated from *t*_1/2_ = 0.693/k_DISS_, and their uncertainties (95% CI) are also reported in Table 2. The 95% CI limits range from 83% to 126% of the central *t*_1/2_ value (Water 1, 1986–2016 GM concentrations) for α-HCH and 89% to 114% of the central *t*_1/2_ value for γ-HCH.

Relative uncertainties in F_OUT_ and F_HYD_ are small, 12% and 16%, respectively. Sedimentation has high uncertainty due to lack of data concerning sediment concentrations and high variability in sedimentation rates (Table SI-2.5); but even using a high sedimentation rate gives a predicted F_SED_ that is negligible compared to other processes. Due to uncertainties in the Whitman two-film model and estimation of K_OL_, the relative error in F_VOL_ could be in the range 50–130% (SI-2.7). Enantioselective degradation of α-HCH (Figure SI-1.4) is a clear indication of microbial degradation, but large uncertainty in the rate constants (Table SI-2.7) makes this process difficult to quantify.

F_LOSS_ sets an upper boundary for the individual process rates. The fact that F_MIC1_ exceeds F_LOSS_ for α-HCH indicates that the *k*_m_ derived from ocean data is too high (SI-2). Process Sum 3 (F_VOL_ + F_OUT_ + F_HYD_ + F_MIC2_) is 174% of F_LOSS_, and it is likely that F_MIC2_ and/or F_VOL_ are overestimated. Because of such limitations, it is better to consider the relative magnitude of removal processes rather than their absolute magnitudes. These are F_VOL_ ≈ F_MIC_ > F_HYD_ > F_OUT_ > F_SED_.

Another long-term data set for HCHs in a water body is a 40-year record from the Arkona Basin of the Baltic Sea, where total HCHs declined from 12.5 ng L^–1^ in 1975 to <0.4 ng L^–1^ in 2015.^64^ Like LS, inputs to the Baltic were largely atmospheric and the decline over 40 years was due to reduction in air concentrations and dissipation processes. Degradation in the Baltic was estimated at two deep-water stations, where a salinity gradient isolates the water from atmospheric exchange. The halving times calculated from observed losses in deep water ranged from 4.7 to 4.9 y for α-HCH, 3.9 to 4.2 y for γ-HCH, and 7.2 to 13.6 y for β-HCH. The isomer proportion has changed from α-HCH > γ-HCH in the 1970s to dominance of β-HCH (measured since 2000) in recent years.

HCHs have declined significantly in the air, water, and fish of LS, a tribute to the success of regulatory controls. It is interesting that the long-term record of γ-HCH in water (Figure 3) shows no irregularities following the lindane phase-out in the U.S. and Canada in the 2000s decade or following the Stockholm Convention ban in 2009. It may be that the near-decade response time for LS water and the low frequency of sampling smooth any “blips” that might have occurred. The discrepancies between the directly measured F_LOSS_ and the sum of loss processes highlights the difficulties in obtaining mass balances based on predictions. Long-term monitoring data are sparse for Great Lakes water. Carlson et al.^7^ stated, “As concentrations in fish reflect concentrations in water, the change in source functions could be the primary factor behind rate changes observed in fish.”

## Supporting Information Available

Collection, analytical methods, and HCH concentrations determined by different research teams; total measured dissipation of α-HCH and γ-HCH from 1986 to 2016; process parameters and estimated losses by volatilization, outflow, sedimentation, hydrolysis and microbial degradation; water concentrations used to estimate volatilization loss in this study and earlier reports; calculation of volatilization mass transfer coefficients; estimation of uncertainties; map of LS with surveillance stations; regressions of the natural logarithms of C_W_/ng L^–1^ (annual AM, annual GM and all points) vs year; enantiomer fractions (EFs) of α-HCH (PDF)
