# Supplementary material for: Lake Superior Has Lost over 90% of Its Pesticide HCH Load since 1986
Source: Environ Sci Technol. 2021 Apr 7;55(14):9518–26. doi: 10.1021/acs.est.0c07549 (PMC8296669; doi:10.1021/acs.est.0c07549)
Supplement: Supplementary file 1 — es0c07549_si_001.pdf [file es0c07549_si_001.pdf]

Lake Superior has lost over 90% of its pesticide HCH load since 1986

Supporting Information

Terry F. Bidleman[1*], Sean Backus[2], Alice Dove[3]

Rainer Lohmann[4], Derek Muir[5], Camilla Teixeira[5], Liisa Jantunen[6]

1. Department of Chemistry, Umeå University, Umeå, SE-901 87, Sweden.
2. Great Lakes Ecosystem Management Section, Environment and Climate Change Canada, Burlington, Ontario L7R 4A6, Canada.
3. Water Quality Monitoring and Surveillance Division, Environment and Climate Change Canada, Burlington, Ontario L7R 4A6, Canada.
4. Graduate School of Oceanography, University of Rhode Island, Narragansett, Rhode Island 02882, USA
5. Aquatic Contaminants Research Division, Environment and Climate Change Canada, Burlington, Ontario L7R 4A6, Canada.
6. Air Quality Processes Research Section, Environment and Climate Change Canada, Egbert, Ontario L0L 1N0, Canada.

Item	Title	Page
SI-1	HCH concentrations in Lake Superior water and enantiomer fractions (EFs) of α-HCH.	S2
SI-2	Calculation of inflows and outflows.	S2
SI-3	References.	S9
Figure SI-1.1	Map of Lake Superior showing surveillance stations.	S13
Figure SI-1.2	Natural logarithms of annual arithmetic mean (AM) and annual geometric mean (GM) concentrations versus year.	S14
Figure SI-1.3	Natural logarithm of individual sample concentrations versus year.	S15
Figure SI-1.4	EFs of α-HCH in Lake Superior surface and deep water.	S16
Table SI-1.1	Collection and analytical methods, HCH concentrations (ng L^{-1}) and EFs of α-HCH in Lake Superior surface water.	S17
Table SI-2.1a	Loss budget of α-HCH from Lake Superior.	S19
Table SI-2.1b	Loss budget of γ-HCH from Lake Superior.	S20

Table SI-2.2	Water concentrations of HCHs used for volatilization calculations in IADN loadings reports and in this study.	S21
Table SI-2.3	Calculation of mass transfer coefficients.	S22
Table SI-2.4	Annual outflow of water and HCHs from the St. Mary's River.	S23
Table SI-2.5	Data for estimating HCH sedimentation flows in Lake Superior.	S24
Table SI-2.6	Hydrolysis of α-HCH and γ-HCH in Lake Superior water.	S25
Table SI-2.7	Microbial degradation rate constants.	S26

SI-1. HCH concentrations in LS water and enantiomer fractions of α-HCH

Figure SI-1.1 shows a map of LS with surveillance stations. Most water samples were collected at some or all of these stations (varied with year). Table SI-1.1 summarizes collection, analytical methods and HCH concentrations in LS surface water, including spring and summer campaigns and collections by different research teams. Average annual geometric mean (GM) concentrations are reported in Table 1, with regressions in Figure 3. Figure SI-1.2 compares regressions of annual AM and GM concentrations and Figure SI-1.3 shows regressions of all concentration points for all samples.

The two enantiomers of the chiral compound α-HCH were determined for water samples collected in 1996-1997, 2001, 2005, 2008 and 2011, using GC columns with chiral stationary phases that reversed the enantiomer elution order[1]. The columns and elution orders were Betadex-cst (BDEXcst), 30 m x 0.25 mm, 0.25 µm film (Restek, Bellefonte, PA, U.S.A.), elution order (+), (–). BGB-172, 30 or 15 m, 0.25 mm, 0.25 µm film (BGB Analytik AG, Switzerland), elution order (–), (+). EFs are reported in Table 1. Decreasing EFs with year and lack of trend with depth are shown in Figure SI-1.4.

SI-2. Calculations of outflows and inflows.

Processes which account for loss of HCHs from LS are presented as mass flows (kg y^{-1}):

$$F_{LOSS} = F_{VOL} + F_{OUT} + F_{SED} + F_{HYD} + F_{MIC} \quad (1)$$

F_{LOSS} is the observed total loss (SI-2.1). Loss processes are volatilization (F_{VOL}), outflow through the St. Mary's River (F_{OUT}), sedimentation (F_{SED}), basic hydrolysis (F_{HYD}) and microbial degradation (F_{MIC}). HCHs have also been added to the lake over time, mainly from atmospheric processes (gas exchange and precipitation). Gains were summarized between 1992 and 2015 by Guo et al.[2] and are not discussed here.

SI-2.1. Total loss (F_{LOSS})

Dissipation of HCHs from LS by all processes (F_{LOSS}, kg y^{-1}) was followed by plotting the natural logarithm of annual GM water concentrations (C_W) versus year (Figure 3), and C_W (kg m^{-3}) was multiplied by lake volume (1.21 x 10^{13} m^3) to obtain quantities in the lake (Q_W, kg). Annual F_{LOSS} of α-HCH and γ-HCH are reported in Table SI-2.1a and Tables SI-2.1a,b.

The quantities of α-HCH and γ-HCH in the lake at the beginning of each year were subjected to individual loss processes (eq 1) to determine their annual contribution to F_{LOSS}.

$$Q_W = Q_W^o \, exp(-k_p t) \qquad (2)$$

$$Q_{W,P} = Q_W^o - Q_W \qquad (3)$$

Q_W and Q_W^o are the final and initial quantities of HCHs in the lake during a particular year (t = 1), $Q_{W,P}$ is the quantity dissipated through a particular process in that year (volatilization, hydrolysis, or microbial degradation), and k_p is the pseudo first-order rate constant for that process. This method was used to calculate annual F_{VOL}, F_{HYD} and F_{MIC}.

SI-2.2 Volatilization (F_{VOL})

Annual volatilization flows[2] were calculated from eq 3:

$$F_{VOL} = A \times C_W \times K_{OL} \qquad (3)$$

F_{VOL} is the volatilization flow (kg y^{-1}), A is the area of LS (8.21 x 10^{10} m^2), C_W (kg m^{-3}) is the annual GM HCH concentration in surface water (Figure 3, Table SI-2.2), and K_{OL} (m y^{-1}) is the

overall mass transfer coefficient considered from the water (liquid) side, which includes resistance to gas transfer in the water and air phases[2,3-5].

$$K_{OL} = \left[{}^{1}\!/_{k_L} + {}^{RT_W}\!/_{Hk_G} \right]^{-1} \qquad (4)$$

In eq 4, k_L and k_G (m y^{-1}) are the mass transfer coefficients for the water (liquid) and air (gas) films, T_W (K) is the surface water temperature, R = 8.31 Pa m^3 mol^{-1}, and H is the Henry's law constant of the HCH compound (Pa m^3 mol^{-1}). Thermodynamically consistent "final adjusted values" (FAVs), as functions of T_W, were used for the Henry's law constants of the HCHs[6]. For C_W, we used annual GM HCH concentrations in surface water from 1986 to 2016 (Figure 3, Table SI-2.2).

K_{OL} varies with temperature through the Henry's law constant, and the individual mass transfer coefficients k_L and k_G, which are functions of wind speed[2-5]. Annual wind speeds and surface water temperatures from 2010 – 2016 ranged between 4 – 8 m s^{-1} and 273 – 293 K, with little difference among these years[2]. We used this range of wind speeds and water temperatures in calculating k_L, k_G and K_{OL} (Table SI-2.3). The resulting K_{OL} varied between 4.8 to 44 m y^{-1} (GM 14 m y^{-1}) for α-HCH and 2.0 to 19 m y^{-1} (GM 6.1 m y^{-1}) for γ-HCH (Table SI-2.3), and the GM K_{OL} values were used in F_{VOL} estimates.

For comparison, K_{OL} values were also estimated from the F_{VOL} results of Guo et al.[2] for years 1992 – 2015, using eq 3 together with HCH water concentrations taken from IADN-GLB reports and published papers (Table SI-2.2). These water concentrations varied inconsistently over several years and often the same value was assumed for multiple years. For example, α-HCH concentrations reported in 1992 – 1996 were actually lower than those in 1997 – 2000. The K_{OL} values used by Guo et al.[2] were calculated for years 1992 – 2015, using the literature values for C_W, and averaged 12.0 ± 6.9 m y^{-1} for α-HCH and 7.8 ± 2.7 m y^{-1} for γ-HCH. These are 14% lower and 28% higher than the GM K_{OL} calculated from "first principles" in the preceding paragraph.

SI-2.3. Outflow (F_{OUT})

The only substantial outflow from LS is the St. Mary's River, which connects LS and Lake Huron. Outflows of α- and γ-HCHs through the St. Mary's River over time were calculated

from the annual GM C_W in LS for a particular year, assuming uniform distribution with depth (Figure 2), and the annual outflow (Table SI-2.4). Annually averaged water discharge rates were calculated from monthly discharge rates[7] Annual discharge rates varied from 1491 – 2606 m^3 s^{-1} from 1986 – 2010, and the decline over these years was significant at $p = 0.035$ (Table SI-2.4). The average discharge 1829 m^3 s^{-1} from 2005 – 2010 was assumed for years 2011 – 2016.

SI-2.4. Sedimentation (F_{SED})

Concentrations of α-HCH and γ-HCH in a sediment core from Jackfish Bay, LS (48°47'49", 86°59'31") averaged 0.087 ± 0.040 and 0.071 ± 0.044 ng g^{-1} dry wt. in 0.5-cm slices dated from 1986 to 1998[8]. Variations were small (0.05 – 0.15 ng g^{-1} for α-HCH and 0.03 – 0.14 ng g^{-1} for γ-HCH) and were slightly higher in 1986 – 1989 slices than those in 1991 – 1998. Lindane (γ-HCH) in open lake surficial sediments was <0.05 ng g^{-1} dry wt. at the 75th percentile in 2001[9]. Reported sedimentation rates in LS ranged from 0.0044 to 1.05 g m^{-2} d^{-1} (Table SI-2.5), and a high value of 1.0 g m^{-2} d^{-1} was assumed to estimate annual sedimentation of 2.6 and 2.1 kg y^{-1} of α-HCH and γ-HCH.

SI-2.5. Hydrolysis (F_{HYD})

The α-HCH and γ-HCH isomers are subject to basic hydrolysis in the slightly alkaline water of the Great Lakes, whereas β-HCH is stable even in strongly basic solution[10]. The second-order and pseudo first-order rate equations are:

$$ln\left(C/_{C^o}\right) = -(k_B \times a_{OH}) \times t \qquad (5)$$

$$ln\left(C/_{C^o}\right) = -k' \times t \qquad (6)$$

Hydrolysis was evaluated in one-year steps. C^o is the concentration at the beginning of the year and C is the concentration remaining at the year's end. This C becomes C^o in the following year. The second-order basic hydrolysis rate constant is k_B (M^{-1} min^{-1}), a_{OH} is the

activity of hydroxyl ion and k' = k_B*a_{OH}. Temperature-dependent k_B for the labile HCHs have been reported[10]:

$$log\ k_{B,\alpha-HCH} = 14.151 - {4091}/{T} \quad (7)$$

$$log\ k_{B,\gamma-HCH} = 15.111 - {4417}/{T} \quad (8)$$

At 278 K, k_B (α-HCH) = 0.272 M^{-1} min^{-1} and k_B (γ-HCH) = 0.167 M^{-1} min^{-1}. The dissociation constant for water follows eq (9)[11]; At the mean temperature of LS (278 K) pK_W = 14.738.

$$Log\ K_W = a_H \times a_{OH} = 6.0875 - {4471}/{T} - 0.01706T \quad (9)$$

The *in situ* pH of western LS surface water, measured from 2014 – 2016, varied with temperature[12]:

$$pH = 0.0135 \times temp, C + 7.8407 \quad r^2 = 0.42 \quad (10)$$

From eq (9) and (10), the pH for surface water at 278 K (5°C) is 7.91. The pH decreases with depth in spring-fall, when stratification removes deeper water from contact with the atmosphere. The *in situ* pH of water at 60 – 210 m averaged 7.83 ± 0.06 (n = 31) from May – October[12]. We selected pH 7.83 (pOH = 14.74 – 7.83 = 6.91) for hydrolysis calculations (Table SI-2.6).

SI-2.6 Microbial degradation (F_{MIC})

EFs of α-HCH in Lake Superior surface water declined linearly from 1996 to 2011 (r^2 = 0.94), and in 2005 the EFs did not vary with depth (Figure SI-1.4). The ratio of pseudo first-order microbial degradation rate constants was k_{m+}/k_{m-} = 1.33 (Table SI-2.7). It is not possible to derive absolute rate constants from this ratio and F_{LOSS} of total α-HCH (sum of enantiomers), because F_{LOSS} involves processes other than degradation (eq 1).

Rate constants for HCH microbial degradation have been reported in the Barents Sea – Eastern Arctic Ocean (EAO)[13,14]: k_{m+} = 0.117 y^{-1} , k_{m-} = 0.030 y^{-1} , k_m = 0.147 y^{-1} (total α-HCH,

sum of enantiomers) and k_m = 0.037 y^{-1} for γ-HCH (Table SI-2.7). The ratio k_{m+}/ k_{m-} = 3.97. For application to Lake Superior, we assumed the marine rate constant k_m = 0.037 y^{-1} for γ-HCH[13,14] (F_{MIC}). Two rate constants were applied for total α-HCH (sum of enantiomers). F_{MIC1}: We used the Barents – EAO k_m = 0.147 y^{-1}; F_{MIC2}: We used the Barents – EAO rate constant for (–)α-HCH (k_{m-} = 0.030 y^{-1})[13,14] and k_{m+} = 0.040 y^{-1} was calculated for (+)α-HCH based on our measured ratio of k_{m+}/ k_{m-} = 1.33. Thus, k_m = 0.030 + 0.040 = 0.070 y^{-1} for total α-HCH (Table SI-2.7).

SI-2.7. Uncertainties in the loss terms.

Uncertainty in F_{LOSS}

The 95% confidence intervals (CI)[15] for k_{DISS} were derived from the standard error (SE) by $t_{0.05,\, n-2}$*SE and are reported in Table 2 of the main paper. Corresponding halving times, calculated from $t_{1/2}$ = -0.693/k_{DISS}, and their uncertainties (95% CI) are also reported in Table 2. The 95% CI limits range from 83% to 126% of the central $t_{1/2}$ value (Water 1, 1986 – 2016 GM concentrations) for α-HCH and 89% to 114% of the central $t_{1/2}$ value for γ-HCH.

Uncertainty in F_{VOL}

Calculation of F_{VOL} involves the annual GM C_W, A (lake area) and K_{OL} (eq 3). Uncertainties in F_{VOL} are mainly associated with those in K_{OL}, which are much higher than for C_W. K_{OL} is a function of the temperature-dependent Henry's law constant (H) and the individual mass transfer coefficients (MTCs) for air and water (k_G, k_L) (eq 4). Uncertainties in H are low; thermodynamically consistent final adjusted values (FAVs) were 87% of average literature-derived values (LDV) for α–HCH and 115% for γ–HCH[6].

The MTCs are nonlinear functions of wind speed and we took the average of MTCs calculated at 4 and 8 m s^{-1}. Our K_{OL} values were 14-28% different from those calculated by Guo et al.[2], who used monthly averaged wind speeds (SI-2.2). Gas exchange of HCHs is limited almost entirely by air-side resistance, so uncertainties in k_G contribute more than k_L to overall uncertainty. If water resistance is neglected, a simplified version of eq (4) is:

$$K_{OL} \approx {}^{Hk_G}\!/_{RT_w} \qquad (12)$$

Thus, uncertainty in K_{OL} are mainly due to that in k_G. A common expression for the k_G of water vapor is:

$$k_G = (0.2U_{10} + 0.3) \qquad (13)$$

where k_G has units of cm s^{-1} and U_{10} (m s^{-1}) is wind speed at 10 m height[4]. Eq 13 is multiplied by the ratio of HCH/water vapor diffusion coefficients ($D_R^{0.61}$) to obtain k_G for HCHs[2] (Table SI-2.3). Rowe and Perlinger[5] noted a factor of two discrepancy between exchange of water vapor modeled by eq 13 and micrometeorological measurements of water vapor exchange. They also found up to 53% difference in K_{OL} estimated by two models, the Whitman two-film (used here) and the Coupled Ocean–Atmosphere Response Experiment (COARE) bulk algorithm. Hoff et al.[16] estimated an uncertainty of 50 – 130% in volatilization fluxes of POPs, about 15-30% of which was due to uncertainty in C_W. Khairy et al.[17] estimated propagated uncertainties of 56 – 131% in air-water exchange fluxes of OCPs. It seems reasonable that uncertainties in F_{VOL} could be 50-130%.

Uncertainty in F_{OUT}

The St. Mary's river discharge data for each year[7] consisted of monthly flow rates, from which we calculated annual mean flow rates. No uncertainties were associated with the monthly flow rate data. The relative standard deviation of the annual mean flow rates during 1986 – 2010 was 11.9% and we took 12% as the uncertainty in F_{OUT}.

Uncertainty in F_{HYD}

Average RSDs of basic hydrolysis rate constants (k_B) over the temperature range 5 – 45 ^oC were 6.9% and 9.1% for α-HCH and γ-HCH, here assumed 8% overall[10]. We selected pH 7.83 ± 0.06 in the 60 –210 m depth range; the pH in surface water tends to be higher and seasonally variable (Section SI-2.5). The $a_{H+} = 10^{-pH}$ has the form
$x = ay^{\pm bu}$, for which a and b are = 1, y = 10, and the relative uncertainty $\sigma_x/x = (\ln 10)*\sigma_u$). The relative uncertainty in a_{H+} (and a_{OH-}) is 2.303*0.06 = 0.138. The pseudo first-order hydrolysis rate constant $k' = k_B \times a_{OH-}$. The RSD (k') = $(RSD_{kb}^2 + RSD_{aOH-}^2)^{0.5} = (0.08^2 + 0.138^2)^{0.5} = 0.16$. The k' are 0.0177 ± 0.00283 for α-HCH and 0.01085 ± 0.00174 for γ-HCH.

Uncertainty in F_{MIC}

Uncertainty in microbial degradation rates was discussed in the main paper, Section 3.3.5. Pseudo first-order rate constants derived from measurements in oceans and lakes varied from 0.147 – 1.13 y^{-1} for total α-HCH (sum of enantiomers) and 0.037 – 0.167 y^{-1} for γ-HCH (Table SI-2.7). Some rate constants are clearly unsuitable, because they imply faster degradation than the measured 0.122 y^{-1} rate constant for F_{LOSS} (Table 2, GM Water 1) Relative rates of α-HCH enantiomer degradation (k_{m+}/k_{m-}) were 3.9 in the Bering Sea – Eastern Arctic Ocean[13,14] and 1.33 measured in LS. The rate constant for (+)α-HCH degradation was derived from this ratio and 0.030 y^{-1} reported for (–)HCH[13,14]. Given these limitations, it is not feasible to set numerical limits on microbial degradation uncertainty, except that realistic rate constants cannot exceed 0.122 y^{-1}.

SI-3. References

1. Jantunen, L.M.; Helm, P.A.; Ridal, J.J.; Bidleman, T.F. Air-water gas exchange of chiral and achiral organochlorine pesticides in the Great Lakes. *Atmos. Environ*. **2008**, *42*, 8533-8542.

2. Guo, J.; Salamova, A.; Venier, M.; Dryfhout-Clark, H.; Alexandrou, N.; Backus S.; Bradley, L.; Hung, H.; Hites, R.A. Atmospheric flows of semi-volatile organic pollutants to the Great Lakes estimated by the United States Integrated Atmospheric Deposition and Canada's Great Lakes Basin Monitoring and Surveillance Networks. *J. Great Lakes Res.* **2018**, *44*, 665-677.

3. Galarneau, E.; Audette, C.V.; Bandemehr, A.; Basu, I.; Bidleman, T.F.; Brice, K.A.; Burniston, D.A.; Chan, C.H.; Froude, F.; Hites, R.A.; Hulting, M.L.; Neilson, M.; Orr, D.; Simcik, M.F.; Strachan, W.M.J.; Hoff, R.M. Atmospheric deposition of toxic substances to the Great Lakes: IADN results to 1996. Environment Canada and the United States Environmental Protection Agency, Public Works and Government Services Canada Catalogue Number: En56-156/2000E-IN, US EPA Report Number: EPA 905-R-00004, ISBN: 0-662-29005-4.

4. Hornbuckle, K.C.; Jeremiason, J.D.; Sweet, C.W.; Elsenreich, S.J. Seasonal variations in air-water exchange of polychlorinated biphenyls in Lake Superior. *Environ. Sci. Technol.* **1994**, *28*, 1491-1501.

5. Rowe, M.D.; Perlinger, J.A. Micrometeorological measurement of hexachlorobenzene and polychlorinated biphenyl compound air-water gas exchange

in Lake Superior and comparison to model predictions. *Atmos. Chem. Phys*. **2012**, *12*, 4607–4617.

6. Xiao, H.; Li, N.; Wania, F. Compilation, evaluation, and selection of physical-chemical property data for α-, β-, and γ-hexachlorocyclohexane. *J. Chem. Eng. Data* **2004**, *49*, 173-185.

7. Noorbakhsh, N., Great Lakes Connecting Channel flows, updated 2010. . https://www.glerl.noaa.gov/ftp/publications/tech_reports/glerl-083/UpdatedFiles/. Accessed March 19, 2021.

8. Muir, D.C.G., personal communication. Environment and Climate Change Canada.

9. Gewurtz, S.; Shen, L.; Helm, P.A.; Waltho, J.; Reiner, E.J.; Painter, S.; Brindle, I.D.; Marvin, C.H. Spatial distributions of legacy contaminants in sediments of lakes Huron and Superior. *J. Great Lakes Res*. **2008**, *34*, 153-168.

10. Ngabe, N.; Bidleman, T.F.; Falconer, R.L. Base hydrolysis of α- and γ-hexachlorocyclohexanes. *Environ. Sci. Technol.* **1993**, *27*, 1930-1933.

11. Harned, H.S.; Owen, B.B. *The Physical Chemistry of Electrolyte Solutions*. Van Nostrand Reinhold, New York, 1958.

12. Minor, E.C., Tennant, C.J., Brown, E.T. A seasonal to interannual view of inorganic and organic carbon and pH in western Lake Superior. *J. Geophys. Res. Biogeosci*. **2019**, *124*, 405–419.

13. Harner, T.; Kylin, H.; Bidleman, T.F.; Strachan, W.M.J. (1999). Removal of α- and γ-hexachlorocyclohexanes (HCHs) and enantiomers of α-HCH in the eastern Arctic Ocean. *Environ. Sci. Technol*. **1999**, *33*, 1157-1164

14. Harner, T.; Jantunen, L.M.; Bidleman, T.F.; Macdonald, R.W.; Kylin, H.; Strachan, W.M.J. Microbial degradation is a key elimination pathway of hexachlorocyclohexanes from the Arctic Ocean. *Geophys. Res. Lett*. **2000**, *27*, 1155-1158.

15. Salamova, A.; Venier, M.; Hites, R.A. Revised temporal trends of persistent organic pollutant concentrations in air around the Great Lakes. *Environ. Sci. Technol. Lett*. **2015**, *2*, 20-25.

16. Hoff, R.M.; Strachan, W.M.J.; Sweet, C.W.; Chan, C.H.; Shackleton, M.; Bidleman, T.F.; Brice, K.A.; Burniston, D.A.; Cussion, S.; Gatz, D.; Schroeder, W.H. Atmospheric deposition of toxic chemicals to the Great Lakes: review of data through 1994. *Atmos. Environ*. **1996**, *30*, 3505-3527.

17. Khairy, M.; Muir, D.; Teixeira, C.; Lohmann, R. Spatial trends, sources, and air−water exchange of organochlorine pesticides in the Great Lakes Basin using low density polyethylene passive samplers. *Environ. Sci. Technol*. **2014**, *48*, 9315-9324.

18. Stevens, R.J.J.; Neilson, M.A. Inter- and intralake distributions of trace organic contaminants in surface waters of the Great Lakes. *J. Great Lakes Res*. **1989**, *15*, 377-393.

19. Dove, A., personal communication. Environment and Climate Change Canada.

20. Williams, D.J.; Kuntz, K.W.; L'Italien, S.; Richardson, V. Organic contaminants in the Great Lakes 1992–1998. Intra- and inter-lake spatial distributions and temporal trends. Ecosystem Health Division, Environmental Conservation Branch, Ontario Region, Burlington, Ontario, Report No. EHD/ECB-OR/01–01/I, 2001.

21. Venier, M.; Dove, A.; Romanak, K.; Backus, S.; Hites, R.A. Flame retardants and legacy chemicals in Great Lakes water. *Environ. Sci. Technol*. **2014**, *48*, 9563-9572.

22. Ruge, Z.; Muir, D.; Helm, P., Lohmann, R. Concentrations, trends, and air−water exchange of PCBs and organochlorine pesticides derived from passive samplers in Lake Superior in 2011. *Environ. Sci. Technol*. **2018**, *52*, 14061-14069.

23. Buehler, S.; Hafner, W.; Basu, I.; Audette, C.V.; Brice, K.A.; Chan, C.H.; Froude, F.; Galarneau, E.; Hulting, M.; Jantunen, L., Neilson, M.; Puckett, K.; Hites, R.A. Hoff, R.M. Atmospheric deposition of toxic substances to the Great Lakes: IADN results through 1998. Environment Canada and the United States Environmental Protection Agency, Public Works and Government Services Canada Catalogue Number: En56-156/1998E, US EPA Report Number: 905-R-01-007, ISBN: 0-662-31219-8.

24. Blanchard, P.; Audette, C.V.; Hulting, M.; Basu, I.; Brice, K.A.; Chan, C.H.; Dryfhout-Clark, H.; Froude, F.; Hites, R.A.; Neilson, M. Atmospheric deposition of toxic substances to the Great Lakes: IADN results through 2000. Environment Canada and the United States Environmental Protection Agency, Public Works and Government Services Canada Catalogue Number: En56-156/2000-1E, US EPA Report Number: 905-R-04-900, ISBN: 0-662-37467-3.

25. Blanchard, P.; Audette, C.V.; Hulting, M.; Basu, I.; Brice, K.A.; Backus, S.M.; Dryfhout-Clark, H.; Froude, F.; Hites, R.A.; Neilson, M.; Wu, R. Atmospheric deposition of toxic substances to the Great Lakes: IADN results through 2005. Environment Canada and the United States Environmental Protection Agency, Public Works and Government Services Canada Catalogue Number: 978-0-662-48287-1, US EPA Report Number: EPA-905-R-08-001, ISBN: 0-662-48287- 5.

26. Baker, J.E.; Eisenreich, S.J.; Eadie, B.J. Sediment trap fluxes and benthic recycling of organic carbon, polycyclic aromatic hydrocarbons, and polychlorobiphenyl congeners in Lake Superior. *Environ. Sci. Technol*. **1991**, *25*, 500-509.

27. Jeremiason, J.D.; Eisenreich, S.J.; Baker, J.E.; Eadie, B.J. PCB decline in settling particles and benthic recycling of PCBs and PAHs in Lake Superior. *Environ. Sci. Technol.* **1998**, *32*, 3249-3256.

28. James, R.R.; McDonald, J.G.; Symonik, D.M.; Swackhamer, D.; Hites, R.A. Volatilization of toxaphene from lakes Michigan and Superior. *Environ. Sci. Technol.* **2001**, *35*, 3653-3660.

29. Muir, D.C.G.; Whittle, D.M.; DeVault, D.S.; Bronte, C.R.; Karlsson, H.; Backus, S.; Teixeira, C. Bioaccumulation of toxaphene congeners in the Lake Superior food web. *J. Great Lakes Res*. **2004**, *30*, 316-340.

30. Li, H.; Minor, E.C. Biogeochemical characteristics of settling particulate organic matter in Lake Superior: A seasonal comparison. *Org. Geochem*. **2015**, *85*, 76-88.

31. Galbán-Malagón, C.J.; Berrojalbiz, N.; Gioia, R-; Dachs, J. The "degradative" and "biological" pumps controls on the atmospheric deposition and sequestration of hexachlorocyclohexanes and hexachlorobenzene in the North Atlantic and Arctic oceans. *Environ. Sci. Technol*. **2013**, *47*, 7195-7203.

32. Helm, P.A.; Diamond, M.L.; Semkin, R.; Bidleman, T.F. Degradation as a loss mechanism in the fate of α-hexachlorocyclohexane in arctic wetlands. *Environ. Sci. Technol*. **2000**, *34*, 812-818.

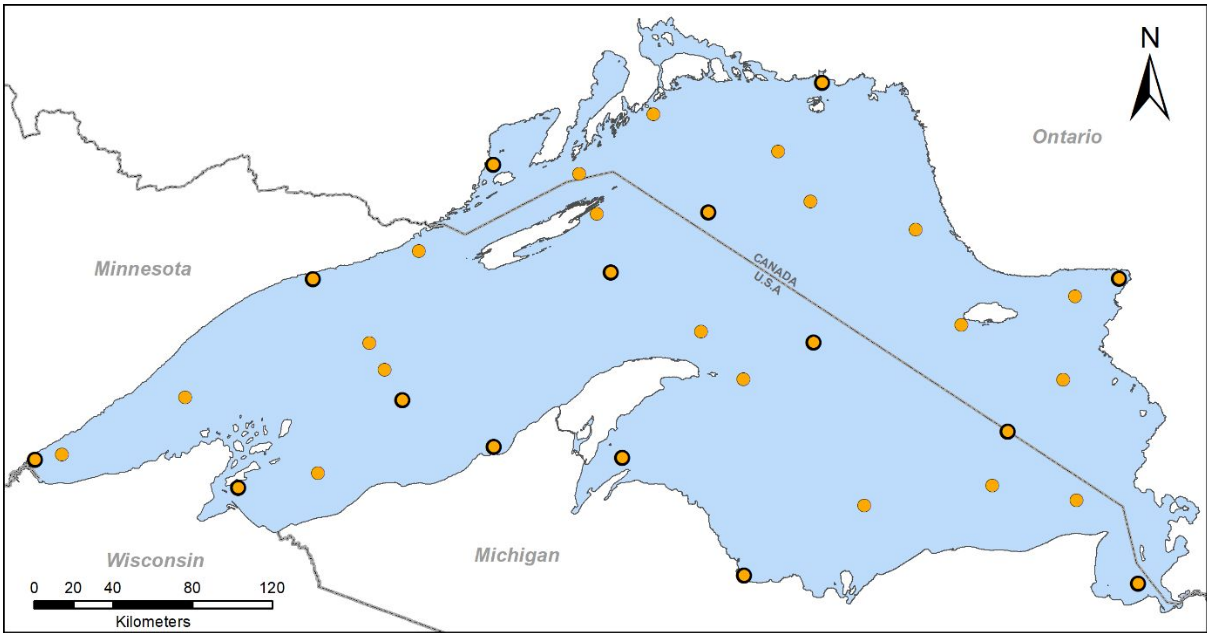

Figure SI-1.1. Great Lakes Surveillance Program stations for water sampling. Most samples were collected at stations with black and orange circles, occasional samples were taken at orange circle stations. Sampling in 2011[21,22] was done at stations shown on maps in their papers.

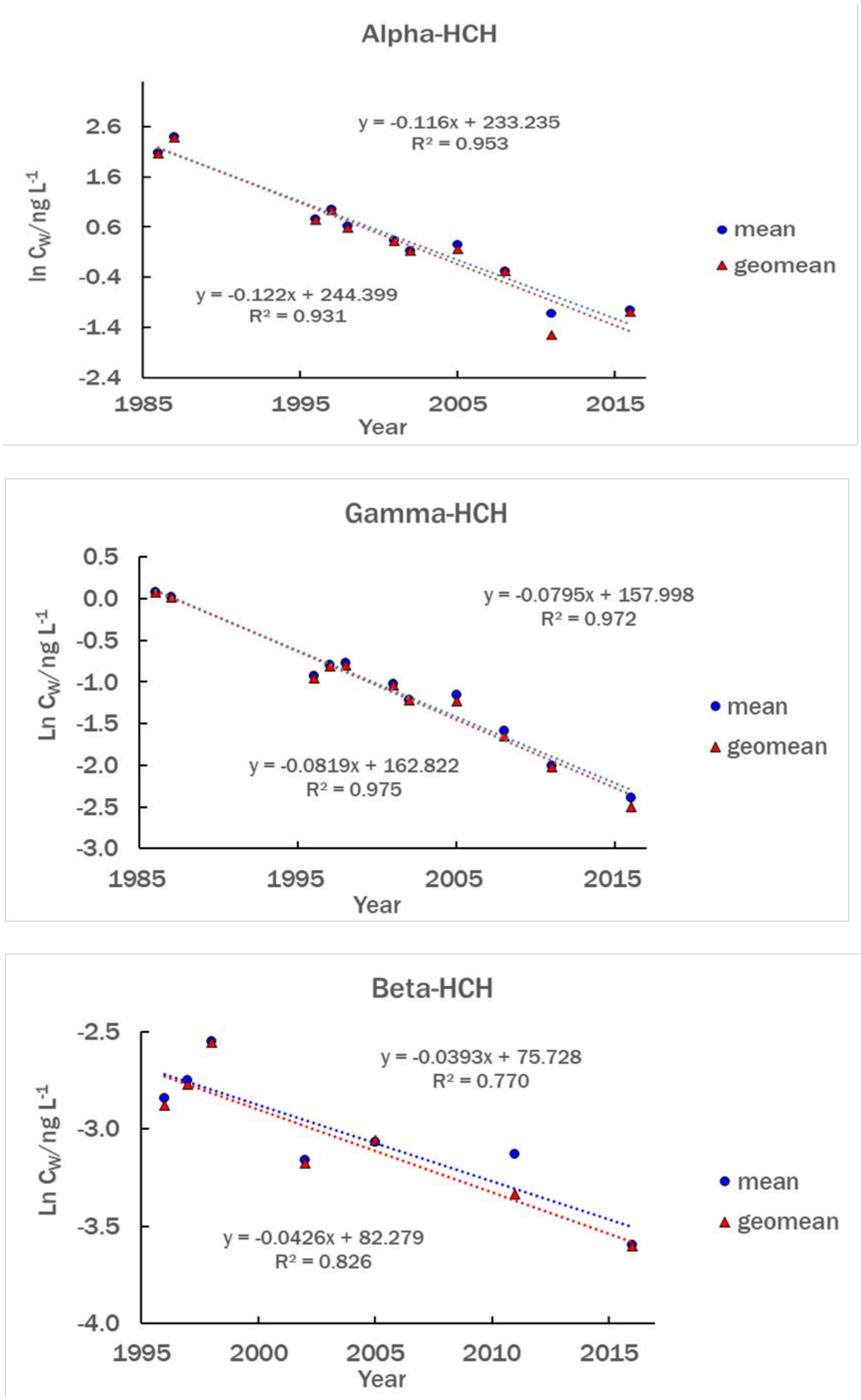

Figure SI-1.2. Natural logarithm of annual arithmetic (AM, blue) and geometric (GM, red) mean concentrations in LS water versus year.

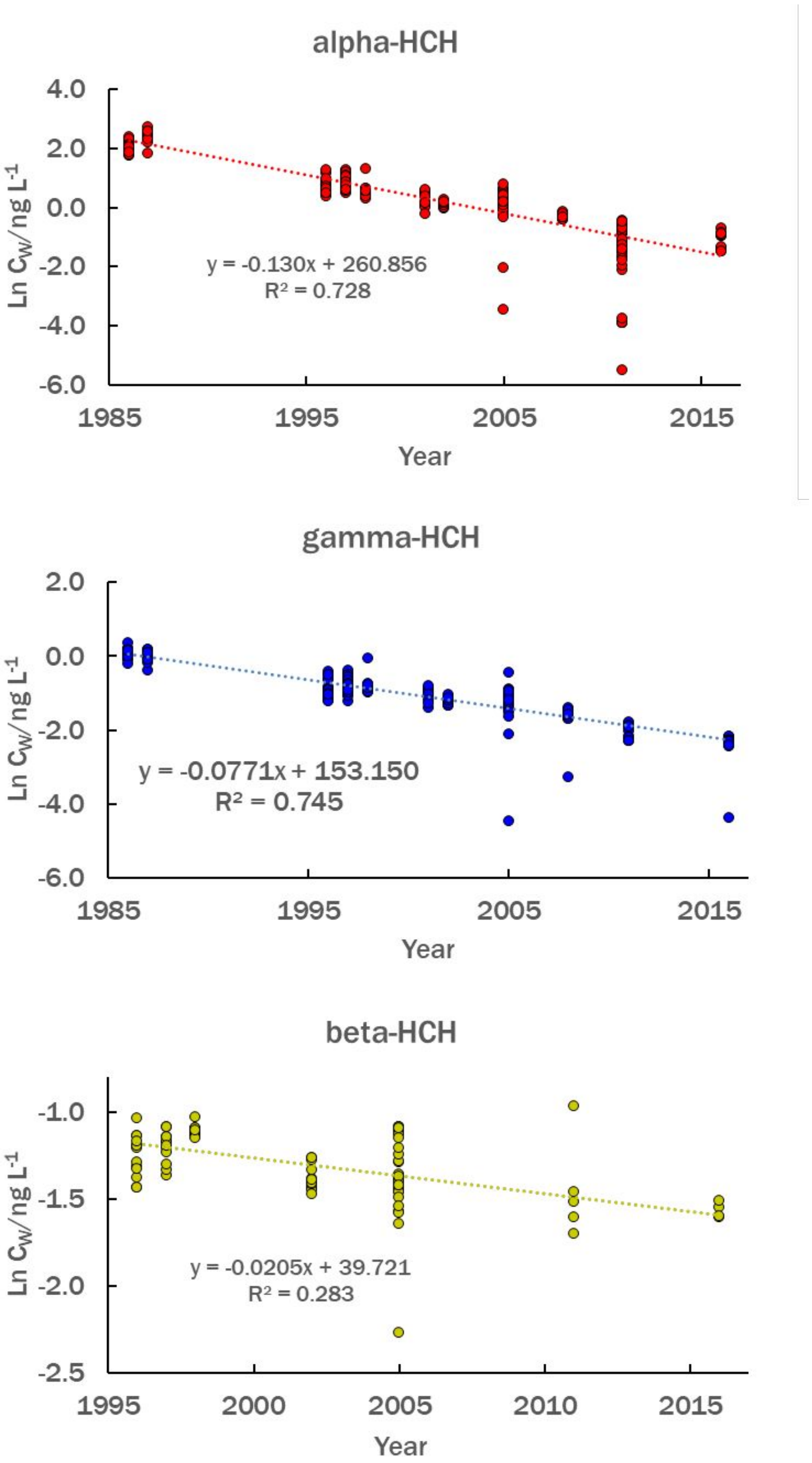

Figure SI-1.3. Natural logarithm of all water sample concentrations in LS versus year.

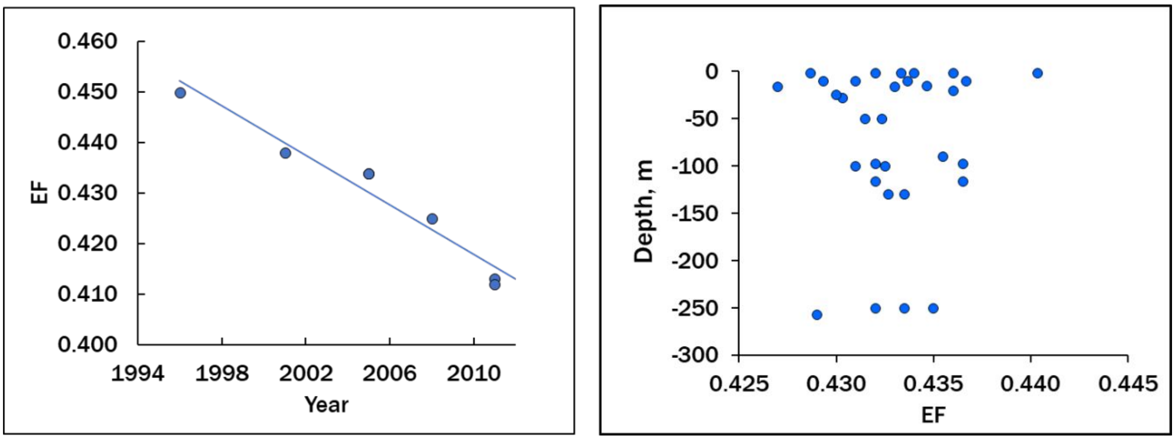

Figure SI-1.4. Left: Enantiomer fractions (EFs) of α-HCH in Lake Superior surface water from 1996 – 1997 to 2011; EF = 5.346 – 0.002452*year, $r^2 = 0.94$. Right: EFs in surface and deep water in August, 2005, no significant trend with depth ($p > 0.05$).

Table SI-1.1. Collection, analytical methods and HCH concentrations (ng L^{-1}) in Lake Superior surface water, as determined by different research teams.

		α-HCH			β-HCH			γ-HCH				Data
Year	Month	Mean	n	SD	Mean	n	SD	Mean	n	SD	Method[a,b]	Source
1986	May	7.98	19	1.33				1.08	19	0.14	1	18,19
1987	May	10.9	19	1.94				1.02	19	0.13	1	18,19
1996	May	2.13	13	0.29				0.36	13	0.03	2	19
1996	August	2.53	5	0.82	0.058	13	0.016	0.58	5	0.05	3	1
1996	August	2.02	14	0.30				0.36	14	0.04	2	19,20
1997	May	2.77	14	0.38	0.064	12	0.013	0.53	14	0.09	3	1
1997	May	2.78	14	0.51				0.38	14	0.04	2	19,20
1997	May	2.16	14	0.27				0.45	14	0.04	2	19,20
1998	May	1.85	17	0.51	0.078	17	0.005	0.46	17	0.13	4	8
2001	May	1.41	14	0.31				0.36	14	0.05	2	19
2002	May	1.14	13	0.09	0.042	13	0.007	0.30	13	0.02	4	8
2005	May	1.25	16	0.35				0.27	16	0.06	5	19
2005	May	1.33	12	0.53	0.033	12	0.011	0.28	12	0.10	4	8
2005	Aug	1.26	17	0.29	0.039	17	0.006	0.34	17	0.02	4	8
2005	Aug.	1.36	11	0.37	0.075	10	0.009	0.37	11	0.11	3	1
2008	May	0.75	18	0.06				0.20	18	0.05	5	19
2011	May	0.47	12	0.09				0.14	12	0.02	5	19
2011	May	0.30	5	0.18	0.044	5	0.037	0.12	4	0.02	6	19,21
2011	June-Oct	0.26	26	0.19							7	22
2016	May	0.42	6	0.04				0.092	7	0.035	5	19
2016	May	0.25	4	0.02	0.027	4	0.003	0.092	4	0.006	8	19

Table SI-1.1. Footnotes.

Vacant spaces: samples not analyzed for this compound.

a) Mean field blanks (ng L^{-1}) for Method 2: α-HCH = 0.009 ± 0.007, γ-HCH = 0.012 ± 0.014.

Field blanks for Method 3: no peaks detected for any HCHs. LOQ for α-HCH and γ-HCH based on the lowest standard was 0.1 ng L^{-1} (4 L). LOQ for β-HCH based on the lowest standard was 0.001 ng L^{-1} (80 L).

Mean field blanks (ng L^{-1}) for Method 5: α-HCH = 0.002 ± 0.001, γ-HCH = 0.004 ± 0.002.

Blanks for Method 7: no peaks detected, LOD estimated at 0.02 ng L^{-1}.

Blanks for Method 8: no peaks detected, LODs were 0.0008 to 0.0011 ng L^{-1} based on 100 L.

No blank information for Methods 4 and 6.

b) Methods:

1. 55 L whole water, extracted onboard ship by large-volume extraction (LVX , Goulden) into DCM, silica gel cleanup, analysis by GC-ECD.
2. 100 L centrifuged and extracted onboard the ship by LVX into DCM, silica gel cleanup, analysis by GC-ECD & GC-MSD.
3. 4 L for α-HCH and γ-HCH (GFF and ENV+® resin) and 80 L for β-HCH (GFF and XAD-2®) analysis by GC-ECNI-MS.
4. 100 L, GFF, XAD-2® resin, analysis by GC-ECD.
5. 24 L filtered onboard, LVX into DCM in a clean lab, silica gel cleanup, analysis by GC-ECD & GC-MSD;
6. 200 L filtered water through XAD-2® resin, analysis by GC-ECD and GC-ECNI-MS.
7. Low density polyethylene (LDPE) passive samplers, GC-MS/MS (Zoe Ruge, Rainer Lohmann)
8. 200 L filtered water through XAD-2® resin, analysis by HRGC/HRMS, SGS AXYS, Canada

Table SI-2.1a. Loss budget for α-HCH in Lake Superior

Year	kg in LS year start	Percent remaining year end	Measured[a] F_{LOSS} kg y^{-1}	F_{VOL} kg/y	F_{OUT} kg/y	F_{HYD} kg/y	F^{b}_{MIC1} kg y^{-1}	F^{c}_{MIC2} kg y^{-1}	F_{PROC1} kg y^{-1}	Process sums[d] F_{PROC2} kg y^{-1}	F_{PROC3} kg y^{-1}
1986	98791	88.5	10624	9585	671	1733	13505	6679	11990	25495	18668
1987	87445	78.3	9404	8485	420	1534	11954	5912	10438	22393	16350
1988	77401	69.4	8324	7510	369	1358	10581	5233	9237	19818	14470
1989	68512	61.4	7368	6648	412	1202	9366	4632	8262	17628	12894
1990	60643	54.3	6521	5884	290	1064	8290	4100	7238	15528	11338
1991	53678	48.1	5772	5208	277	942	7338	3629	6427	13765	10056
1992	47513	42.6	5109	4610	277	834	6495	3212	5721	12216	8933
1993	42056	37.7	4523	4081	240	738	5749	2843	5058	10807	7901
1994	37226	33.4	4003	3612	212	653	5089	2517	4477	9566	6993
1995	32950	29.5	3543	3197	164	578	4505	2228	3939	8444	6167
1996	29166	26.1	3136	2830	197	512	3987	1972	3539	7526	5511
1997	25816	23.1	2776	2505	164	453	3529	1745	3122	6651	4867
1998	22851	20.5	2457	2217	104	401	3124	1545	2722	5846	4267
1999	20227	18.1	2175	1963	104	355	2765	1367	2421	5186	3789
2000	17904	16.0	1925	1737	86	314	2448	1210	2137	4585	3348
2001	15847	14.2	1704	1538	79	278	2166	1071	1895	4061	2966
2002	14027	12.6	1508	1361	77	246	1918	948	1684	3602	2632
2003	12416	11.1	1335	1205	62	218	1697	839	1485	3182	2324
2004	10990	9.8	1182	1066	60	193	1502	743	1319	2821	2062
2005	9728	8.7	1046	944	56	171	1330	658	1171	2501	1828
2006	8611	7.7	926	835	42	151	1177	582	1029	2206	1611
2007	7622	6.8	820	740	30	134	1042	515	903	1945	1418
2008	6746	6.0	725	655	34	118	922	456	807	1729	1263
2009	5972	5.4	642	579	28	105	816	404	713	1529	1116
2010	5286	4.7	568	513	22	93	723	357	628	1350	985
2011	4679	4.2	503	454	19	82	640	316	555	1195	872
2012	4141	3.7	445	402	18	73	566	280	493	1059	773
2013	3666	3.3	394	356	17	64	501	248	437	938	684
2014	3245	2.9	349	315	15	57	444	219	386	830	606
2015	2872	2.6	309	279	13	50	393	194	342	735	536
2016	2542	2.7	273	247	13	45	348	172	304	652	476
Total			90393	81558	4574	14747	114911	56828	100879	215790	157707
% of ΣF_{LOSS}				90.2	5.1	16.3	127.1	62.9	111.6	238.7	174.5

a) Based on regression of ln (geomean C_W) versus year, Figure 3.

b) F_{MIC1} uses k_m for total α-HCH (sum of enantiomers)[13,14] = 0.147 y^{-1}.

c) F_{HMIC2} uses k_m for total α-HCH (sum of enantiomers) = 0.070 y^{-1}, see Section SI-2.6.

d) $F_{PROC1} = F_{VOL} + F_{OUT} + F_{HYD}$; $F_{PROC2} = F_{VOL} + F_{OUT} + F_{HYD} + F_{MIC1}$; $F_{PROC3} = F_{VOL} + F_{OUT} + F_{HYD} + F_{MIC2}$.

Table SI-2.1b. Loss budget for γ-HCH in Lake Superior

Year	kg in LS year start	Percent remaining year end	Measured[a] F_{LOSS} kg y^{-1}	F_{VOL} kg/y	F_{OUT} kg/y	F_{HYD} kg/y	F_{MIC} kg y^{-1}	Process sums[b] F_{PROC1} kg y^{-1}	F_{PROC2} kg y^{-1}
1986	13222	92.1	1040	548	90	143	480	781	1261
1987	12182	84.9	958	505	58	131	442	695	1137
1988	11223	78.2	883	465	54	121	408	640	1048
1989	10340	72.1	813	429	62	112	376	602	978
1990	9527	66.4	749	395	46	103	346	543	889
1991	8777	61.2	691	364	45	95	319	504	823
1992	8087	56.4	636	335	47	87	294	470	763
1993	7451	51.9	586	309	42	80	271	432	702
1994	6864	47.8	540	285	39	74	249	398	647
1995	6324	44.1	498	262	32	68	230	362	592
1996	5827	40.6	458	242	39	63	212	344	555
1997	5368	37.4	422	223	34	58	195	315	510
1998	4946	34.5	389	205	23	53	180	281	461
1999	4557	31.8	359	189	23	49	166	262	427
2000	4198	29.3	330	174	20	45	153	240	392
2001	3868	27.0	304	160	19	42	141	221	362
2002	3564	24.8	280	148	20	38	129	206	335
2003	3283	22.9	258	136	16	35	119	188	307
2004	3025	21.1	238	125	16	33	110	175	284
2005	2787	19.4	219	116	16	30	101	162	263
2006	2568	17.9	202	106	13	28	93	147	240
2007	2366	16.5	186	98	9.2	26	86	133	219
2008	2180	15.2	171	90	11	24	79	125	204
2009	2008	14.0	158	83	10	22	73	114	187
2010	1850	12.9	146	77	7.8	20	67	104	172
2011	1705	11.9	134	71	7.1	18	62	96	158
2012	1571	10.9	124	65	7.0	17	57	89	146
2013	1447	10.1	114	60	6.5	16	53	82	135
2014	1333	9.3	105	55	6.0	14	48	76	124
2015	1228	8.6	97	51	5.6	13	45	70	114
2016	1132	7.9	89	47	5.2	12	41	64	105
Total			12179	6418	830	1671	5623	8919	14542
% of ΣF_{LOSS}				52.7	6.8	13.7	46.2	73.2	119.4

a) Based on regression of ln (geomean C_W) versus year, Figure 3.

b) $F_{PROC1} = F_{VOL} + F_{OUT} + F_{HYD;}$ $F_{PROC2} = F_{VOL} + F_{OUT} + F_{HYD} + F_{MIC}$

Table SI-2.2. Water concentrations of HCHs used for volatilization calculations in IADN-GLB loadings reports and in this study (Figure 3).

	IADN Cw, ng L^{-1}		Data	This study, Cw^{a}, ng L^{-1}	
Year	α-HCH	γ-HCH	Source	α-HCH	γ-HCH
1986				8.16	1.09
1987				7.23	1.01
1988				6.40	0.93
1989				5.66	0.85
1990				5.01	0.79
1991				4.44	0.73
1992	1.1	0.40	16	3.93	0.67
1993	1.1	0.40	16	3.48	0.62
1994	1.1	0.40	16	3.08	0.57
1995	1.90	0.328	3	2.72	0.52
1996	1.90	0.328	3	2.41	0.48
1997	2.04	0.439	23	2.13	0.44
1998	2.04	0.439	23	1.89	0.41
1999	2.04	0.439	23,24	1.67	0.38
2000	2.04	0.439	23,24	1.48	0.35
2001	1.5	0.31	25	1.31	0.32
2002	1.5	0.31	25	1.16	0.29
2003	1.5	0.31	25	1.03	0.27
2004	1.5	0.31	25	0.91	0.25
2005	1.5	0.31	25	0.80	0.23
2006	uncertain	uncertain		0.71	0.21
2007	uncertain	uncertain		0.63	0.20
2008	uncertain	uncertain		0.56	0.18
2009	uncertain	uncertain		0.49	0.17
2010	0.30	0.117	2,21	0.44	0.15
2011	0.30	0.117	2,21	0.39	0.14
2012	0.30	0.117	2,21	0.34	0.13
2013	0.30	0.117	2,21	0.30	0.12
2014	0.30	0.117	2,21	0.27	0.11
2015	0.30	0.117	2,21	0.24	0.10
2016				0.21	0.094

a) Geometric means.

Table SI-2.3. Calculation of mass transfer coefficients at wind speeds 4 to 8 m s^{-1} and water temperatures 273 to 293 K.

Footnote	Parameters	Values
A	V_M	243.6
B	D_R	0.2189
C	log H, α-HCH	$10.26-3099/T_W$
C	log H, γ-HCH	$9.94-3117/T_W$
	wind speed, m s^{-1}	4 to 8

MTC calculations

Footnote	Parameters	Values
D	k_L, cm h^{-1}	$0.45*U_{10}^{1.64}*V_M/29.3)^{-0.3}$
D	k_G, cm h^{-1}	$(0.2U_{10}+0.3)D_R^{0.61}$
E	T_W	273 to 293
	k_L at U_{10} = 4 m s^{-1}	6.432E-06
	k_L at U_{10} = 8 m s^{-1}	2.005E-05
	k_G at U_{10} = 4 m s^{-1}	4.355E-03
	k_G at U_{10} = 8 m s^{-1}	7.521E-03

$$K_{OL} = \left[{}^{1}/_{k_L} + {}^{RT_W}/_{Hk_G}\right]^{-1}$$

K_{OL} at two wind speeds and two temperatures (low-high range)

**U_{10} = 4, T_W = 273**		**U_{10} = 4, T_W = 293**		**U_{10} = 4, T_W = 273**		**U_{10} = 4, T_W = 293**	
H (α-HCH)	0.0810	H (α-HCH)	0.4822	H (γ-HCH)	0.0333	H (γ-HCH)	0.2003
RT_W/Hk_G	6.437E+06	RT_W/Hk_G	1.160E+06	RT_W/Hk_G	1.565E+07	RT_W/Hk_G	2.792E+06
$1/K_{OL}$	6.592E+06	$1/K_{OL}$	1.316E+06	$1/K_{OL}$	1.581E+07	$1/K_{OL}$	2.948E+06
K_{OL}	1.517E-07	K_{OL}	7.601E-07	K_{OL}	6.326E-08	K_{OL}	3.392E-07

**U_{10} = 8, T_W = 273**		**U_{10} = 8, T_W = 293**		**U_{10} = 8, T_W = 273**		**U_{10} = 8, T = 293**	
H (α-HCH)	0.0810	H (α-HCH)	0.4822	H (γ-HCH)	0.0333	H (γ-HCH)	0.2003
RT_W/Hk_G	3.727E+06	RT_W/Hk_G	6.717E+05	RT_W/Hk_G	9.063E+06	RT_W/Hk_G	1.617E+06
$1/K_{OL}$	3.777E+06	$1/K_{OL}$	7.216E+05	$1/K_{OL}$	9.112E+06	$1/K_{OL}$	1.666E+06
K_{OL}	2.648E-07	K_{OL}	1.386E-06	K_{OL}	1.097E-07	K_{OL}	6.001E-07

**Geomean K_{OL}, α-HCH, m s^{-1}**	**4.54E-07**	**Geomean K_{OL}, γ-HCH, m s^{-1}**	**1.94E-07**
**Range: 1.5 to 13.9 x 10^{-7}**		**Range: 0.63 to 6.0 x 10^{-7}**	

A. V_M = molecular volume (cm^3 mol^{-1}). See Table S2 in Guo et al.[2].

B. D_R (unitless) = diffusivity ratio of target compound in air relative to water vapor in air. See Table S2 in Guo et al.[2].

C. H = Henry's law constant, Pa m^3 mol^{-1}, at indicated water temperature[6].

D. Mass transfer coefficients for liquid (water) and gas (air) films in cm h^{-1}, U_{10} is the wind speed at 10 m height (m s^{-1}).

E. Water temperature, K.

Table SI-2.4. Annual outflow of water and HCHs from the St. Mary's River.

Year	$m^3 s^{-1}$ Water[a,b]	C_W[c], kg m^{-3} α-HCH	γ-HCH	Outflow, kg y^{-1} α-HCH	γ-HCH
1986	2606	8.16E-09	1.09E-09	671	89.8
1987	1842	7.23E-09	1.01E-09	420	58.5
1988	1829	6.40E-09	9.28E-10	369	53.5
1989	2309	5.66E-09	8.55E-10	412	62.2
1990	1834	5.01E-09	7.87E-10	290	45.5
1991	1981	4.44E-09	7.25E-10	277	45.3
1992	2238	3.93E-09	6.68E-10	277	47.2
1993	2187	3.48E-09	6.16E-10	240	42.5
1994	2183	3.08E-09	5.67E-10	212	39.0
1995	1912	2.72E-09	5.23E-10	164	31.5
1996	2594	2.41E-09	4.82E-10	197	39.4
1997	2440	2.13E-09	4.44E-10	164	34.1
1998	1749	1.89E-09	4.09E-10	104	22.5
1999	1972	1.67E-09	3.77E-10	104	23.4
2000	1841	1.48E-09	3.47E-10	86	20.1
2001	1920	1.31E-09	3.20E-10	79	19.4
2002	2102	1.16E-09	2.95E-10	76.8	19.5
2003	1927	1.03E-09	2.71E-10	62.3	16.5
2004	2091	9.08E-10	2.50E-10	59.9	16.5
2005	2218	8.04E-10	2.30E-10	56.2	16.1
2006	1892	7.12E-10	2.12E-10	42.5	12.7
2007	1491	6.3E-10	1.96E-10	29.6	9.2
2008	1940	5.58E-10	1.8E-10	34.1	11.0
2009	1821	4.94E-10	1.66E-10	28.3	9.5
2010	1615	4.37E-10	1.53E-10	22.2	7.8
2011[b]	1829	3.87E-10	1.41E-10	22.3	8.1
2012	1829	3.42E-10	1.3E-10	19.7	7.5
2013	1829	3.03E-10	1.2E-10	17.5	6.9
2014	1829	2.68E-10	1.1E-10	15.5	6.4
2015	1829	2.37E-10	1.02E-10	13.7	5.9
2016	1829	2.4E-10	9.53E-11	13.8	5.5

a) Great Lakes Connecting Channel flows[7].
https://www.glerl.noaa.gov/ftp/publications/tech_reports/glerl-083/UpdatedFiles/

b) Reports only through 2010. Flows from 2011 – 2016 were averages of 2005 – 2010 values.

c) Geometric mean concentrations, from regressions in Figure 3.

Table SI-2.5. Data for estimating HCH sedimentation flows in LS.

Sedimentation fluxes[a]

Year	Location	Method	$g\ m^{-2}\ d^{-1}$	Reference
1984 – 1985	central – s. central	traps	0.14 – 0.89	26
1987	central – s. central	traps	0.60	27
1991	central – s. central	traps	1.05	27
1997	south central	cores	0.27	28
1997	central	cores	0.44	28
1997	east central	cores	0.16	28
1998	Jackfish Bay	cores	0.17	29
1998	central	cores	0.25	29
1998	north central	cores	0.27	29
2009 – 2010	east	traps	0.0044 – 0.38 (E)	30
2009 – 2010	west	traps	0.06 – 0.59 (W)	30

Concentrations in sediment, C_{SED} ng g^{-1} dry wt.

		**α-HCH**	**γ-HCH**	
Jackfish Bay	1986 –1998	0.087 ± 0.040	0.071 ± 0.044	8
Open lake	2001		< 0.05	9
**Sedimentation flow[a], kg y^{-1}**		2.6	2.1	

a) Flow (kg y^{-1}) = (C_{SED}, ng g^{-1})*(1 g m^{-2} d^{-1})*(10^{-12} kg ng^{-1})*8.21 x 10^{10} m^2*365 d y^{-1} assumes the Jackfish Bay mean concentrations.

Table SI-2.6. Hydrolysis of α-HCH and γ-HCH in Lake Superior water

	Reference
$log\ k_{B,\alpha-HCH} = 14.151 - {}^{4091}/_{T}$	10
$log\ k_{B,\gamma-HCH} = 15.111 - {}^{4417}/_{T}$	10
$ln({}^{C}/_{C^{o}}) = -(k_B \times a_{OH}) \times t$	
$ln({}^{C}/_{C^{o}}) = -k' \times t$	
where $k' = k_B \times a_{OH}$	
$Log\ K_W = a_H \times a_{OH} = 6.0875 - {}^{4471}/_{T} - 0.01706T$	11

	$L\ mol^{-1}\ min^{-1}$					y^{-1}	
Temp.	$k_{B,\alpha\text{-HCH}}$	$k_{B,\gamma\text{-HCH}}$	K_W	a_H	a_{OH}	$k'_{\alpha\text{-HCH}}$	$k'_{\gamma\text{-HCH}}$
278	0.272	0.167	1.828E-15	1.48E-08	1.24E-07	0.01770	0.01085

Table SI-2.7. Microbial degradation rates constants for HCHs in fresh and salt water.

1. Published pseudo first-order microbial degradation rate constants (k_m) y^{-1} — **Reference**

$C = C^o \exp(-k_m t)$

k_m is the rate constant for γ–HCH or total α–HCH (both enantiomers).
k_{m+} and k_{m-} are the rate constants for (+)α–HCH, and (−)α–HCH.

				Reference
total α–HCH[a]	k_m	0.147	Barents Sea	13, 14
total α–HCH	k_m	0.662[a]	Greenland Sea	31
total α–HCH	k_m	0.48 - 1.13	Amituk Lake, Canadian Arctic	32
(+)α–HCH	k_{m+}	0.117	Barents Sea	13, 14
(−)α–HCH	k_{m-}	0.030	Barents Sea	13, 14
	k_{m+}/k_{m-}	3.97	Barents Sea	
γ–HCH	k_m	0.037	Barents Sea	13, 14
γ–HCH	k_m	0.167[b]	Greenland Sea	31

2. Rate constants for Lake Superior, y^{-1}

			Reference
total α–HCH	k_m	0.070	Derived, see below
(+)α–HCH	k_{m+}	0.040	Derived, see below
(−)α–HCH	k_{m-}	0.030	13, 14
	k_{m+}/k_{m-}	1.33	
γ–HCH	k_m	0.037	13, 14

3. Definitions and derivations

C_+ and C_- are concentrations of (+)α–HCH and (−)α–HCH.
C^o refers to concentrations in 1996, C to concentrations in 2011.

$ER = C_+/C_-$	Enantiomer ratio	$ER = \exp-(k_{m+} - k_{m-})t$	13
$EF = C_+/(C_+ + C_-)$	Enantiomer fraction	$C_+ = C^o_+ {*}\exp(-k_{m+}t)$	
$ER = EF/(1\text{-}EF)$		$C_- = C^o_- {*}\exp(-k_{m-}t)$	
EFs from Figure SI-1.2 regression:		$C_+/C_- = C^o_+/C^o_- {*}\exp[(-k_{m+}t) - (-k_{m-}t)]$	
1996: EF = 0.452, ER = 0.825		$C_+/C_- = C^o_+/C^o_- {*}\exp(-k_{m+} + k_{m-})t$	
2011: EF = 0.415, ER = 0.709		$C_+/C_- = C^o_+/C^o_- {*}\exp-(k_{m+} - k_{m-})t$	
$\ln ER_{1996} = -0.19237$		$ER = ER^o {*}\exp-(k_{m+} - k_{m-})t$	
$\ln ER_{2011} = -0.34390$		$ER_{2011} = ER_{1996} {*}\exp-(k_{m+} - k_{m-})t$	
t = 15 y		$(k_{m+} - k_{m-}) = (\ln ER_{1996} - \ln ER_{2011})/t$	
		$(k_{m+} - k_{m-}) = ((-0.19237) - (-0.34390))/15$ $= 0.010102$	
		Take $k_{m-} = 0.030\ y^{-1}$	13
		$k_{m+} - 0.030 = 0.010102$	
		$k_{m+} = 0.040\ y^{-1}$	
		$k_{m+}/k_{m-} = 1.33$	
		$k_m = 0.030 + 0.040 = 0.070\ y^{-1}$	

a) Deep-water k_m (0.147 y^{-1})[13] scaled to account for 4.5X greater microbial biomass/production in epipelagic water[31].
b) Deep-water k_m (0.037 y^{-1})[13] scaled to account for 4.5X greater microbial biomass/production in epipelagic water[31].
